# CILP1 interacting with YBX1 promotes hypertrophic scar formation by suppressing PPARs transcription

**DOI:** 10.1038/s41419-025-07554-8

**Published:** 2025-05-09

**Authors:** Jianzhang Wang, Juan Du, Yajuan Song, Xiaoying Tan, Junzheng Wu, Tong Wang, Yi Shi, Xingbo Xu, Zhou Yu, Baoqiang Song

**Affiliations:** 1 Department of Plastic Surgery, Xijing Hospital, Fourth Military Medical University (Air Force Medical University), Xi’an, 710032 China; 2 Department of Dermatology, Xuanwu Hospital, Capital Medical University, Beijing, 100053 China; 3 Department of Nephrology and Rheumatology, University Medical Center Göttingen, Robert-Koch-Str. 40, 37075 Göttingen, Germany; 4 Clinic for Cardiology and Pulmonology, University Medical Center Göttingen, Robert-Koch-Str. 40, 37075 Göttingen, Germany

**Keywords:** Cell signalling, Trauma, Mechanisms of disease

## Abstract

Hypertrophic scar (HS) represents the most prevalent form of skin fibrosis, significantly impacting the quality of life. Despite this, the molecular mechanisms driving HS formation remain largely undefined, impeding the development of effective treatments. The study showed that Cartilage Intermediate Layer Protein 1 (CILP1) was predominantly expressed in myofibroblasts and was up-regulated in various forms of skin fibrosis, including human hypertrophic and keloid scars, and in animal models of HS. Notably, we detected elevated serum levels of CILP1 in fifty-two patients with HS compared to twenty healthy individuals, suggesting its potential as a novel biomarker. The findings indicated that CILP1 was involved in a negative feedback loop with TGF-β and inhibited the transcription of Peroxisome Proliferator-Activated Receptors (PPARs) via interaction with Y-box-binding protein 1 (YBX1). This interaction promoted cell proliferation, migration, and collagen production in hypertrophic scar fibroblasts (HSFs). In vivo studies further confirmed that CILP1 knockdown markedly reduced HS formation, whereas administration of recombinant human CILP1 protein exacerbated it. These discoveries illuminated the CILP1-YBX1-PPARs signaling pathway as a key regulator of HS formation, offering a foundation for novel therapeutic approaches.

## Introduction

Hypertrophic scar (HS), the fibro-proliferative skin disease, involves excessive extracellular matrix (ECM) deposition inside the initial wounded region caused by surgery, burn and traumatic injury [1–3]. Clinically, HS shows the typical features of red, protruding, hard, or itchy skins, resulting in physical and psychological diseases. In America, the cost of the treatment of HS reaches at least $12 billion annually. Particularly for developing countries, on account of high incidence rate of burn, trauma, and surgery, the number of hyperplastic scar patients is tremendous and the influence of them on economic and society is immeasurable [4, 5]. The current strategies for HS treatment included pressure therapy, excision, intralesional corticosteroid injections, radiation, laser therapy, interferon therapy and so on. All those treatment strategies are associated with a high recurrence and the occurrence of adverse events, such as postoperative incision scar, dishyperpigmentation after corticosteroid application, and induction of basal cell carcinoma after radiation [6]. Nonetheless, its underlying mechanism remains largely unclear, besides, anti-scar treatment should be further improved. Furthermore, no useful biomarkers are clinically available to predict HS. Therefore, it is valuable and promising to search for the potential biomarkers for HS and elucidate its occurrence mechanism mediated by the explored biomarkers, thus contributing to the targeted therapy of HS.

The mechanism of HS may involve the activation of fibroblasts, specifically myofibroblasts [7]. In the process of wound repair, fibroblasts are activated immediately and differentiate into myofibroblasts, and various pathological events are triggered, such as fibroblast proliferation, excess ECM deposition, wound contraction, and finally HS formation [8]. Multiple attempts targeting to myofibroblasts have been made to treat the aberrant skin wound repair and HS formation [9, 10]. Besides, existing studies demonstrated that various matricellular proteins secreted by myofibroblasts or fibroblasts are involved in fibroblasts activation and remodeling of the extracellular matrix environment, thus contributing to HS formation [11]. In addition, matricellular proteins represent the attractive targets for HS treatment owing to their accessibility to HS [12, 13].

Cartilage intermediate layer proteins (CILPs) are matricellular proteins which were initially discovered predominantly produced by chondrocytes within articular cartilage [14, 15]. CILPs are composed of CILP1 and CILP2, which displayed high similarity in amino acid sequences. Existing studies have revealed the close relationship between CILP1 and fibrosis [16]. The expression of CILP1 is up-regulated in cardiac fibroblasts and CILP1 is the new biomarker that can be used to assess disease severity and detect early maladaptive changes in cardiac fibrosis [17]. Mechanistically, CILP1 serves as the molecule downstream of TGF-β signaling within cardiac fibroblasts, which is related to cardiac fibrosis exacerbation [17]. In addition, CILP1 promoted the unfavorable myocardial fibrosis and cardiac remodeling through facilitating myofibroblast growth by mTOR pathway[18]. Thus, CILP1 has been considered to be an important candidate contributor to fibrosis. However, to our best knowledge, the roles and effects of CILP1 on HS formation have not been reported yet. Further, whether CILP1 can be used as a biomarker for HS is still unclear.

This study is the first to demonstrate the up-regulation of CILP1 in various human and animal skin fibrotic diseases, as well as the elevated levels of CILP1 in patients with HS. We found that CILP1 mediates TGF-β-induced hypertrophic scar formation. Notably, CILP1 knockdown results in enhanced TGF-β expression. Furthermore, CILP1 transcriptionally represses the PPARs signaling pathway through its interaction with YBX1, which in turn stimulates fibroblast activation. Knocking down CILP1 also significantly alleviated HS formation in mice. In summary, CILP1 promotes HS formation through the CILP1-YBX1-PPARs pathway and may serve as a promising serological biomarker for HS. Consequently, targeting CILP1 could offer a viable approach for HS treatment.

## Material and methods

### Ethics statement

Human tissues utilized for the present work were obtained in sixteen patients admitted into the Department of Plastic Surgery of Xijing Hospital. Among these samples, six HS samples and their corresponding normal skins were used for RNA sequencing, five HS samples and their corresponding normal skins were applied for immunohistochemistry staining, immunofluorescence staining, Western blot assay, and cell isolation and culture, five keloid scar (KS) samples and their corresponding normal skins were utilized for immunohistochemistry staining. The normal skin samples were obtained from tissues more than 3 cm distant from the affected lesions or the remaining skin tissues after reconstructive surgeries. The patient information described above was listed in Tables 1–3 separately. Additionally, serum samples from fifty-two HS patients and twenty healthy volunteers were also collected for assessing the concentrations of CILP1 (Table 4). The study was conducted in accordance with the principles of the Helsinki Declaration. All patients were informed of the study purpose and processes and provided the informed consent for participation into the present work. The Ethics Committee of Xijing Hospital affiliated to the Fourth Military Medical University approved the present work (approval number: No.KY20203134-1). Our animal experiments followed the Guide for the Care and Use of Laboratory Animals. Experimental protocols were approved by the Committee on the Ethics of Animal Experiments of Fourth Military Medical University; approval number: No.20230033.

**Table 1:** Characteristics of patients with hypertrophic scar and corresponding normal skin tissues utilized for RNA sequencing.

Patient	Sex	Age (years)	Origin	NS or HS (Duration)	Applications
1	Male	3	Hand	NS	RNA-seq
			Hand	HS (10 months)	
2	Male	35	Tempus	NS	RNA-seq
			Tempus	HS (18 months)	
3	Female	17	Face	NS	RNA-seq
			Face	HS (12 months)	
4	Female	10	Thigh	NS	RNA-seq
			Thigh	HS (24 months)	
5	Male	33	Abdomen	NS	RNA-seq
			Face	HS (36 months)	
6	Female	42	Upper eyelid	NS	RNA-seq
			Abdomen	HS (12 months)	

*HS* hypertrophic scar, *NS* normal skin, *RNA-seq* RNA sequencing.

**Table 2:** Characteristics of patients with hypertrophic scar and corresponding normal skin tissues applied for immunostaining, molecular biological experiments, and cell culture.

Patient	Sex	Age (years)	Origin	NS or HS (Duration)	Applications
1	Male	12	Hand	NS	(1) IHC of CILP1 or PPARs protein expression. (2) WB of CILP1 protein expression. (3) RT-qPCR of CILP1 mRNA expression. (4) IF of CILP1 and α-SMA co-staining. (5) Cell isolation and Culture.
			Hand	HS (12 months)	
2	Female	44	Neck	NS	(1) IHC of CILP1 or PPARs protein expression. (2) WB of CILP1 protein expression. (3) RT-qPCR of CILP1 mRNA expression. (4) IF of CILP1 and α-SMA co-staining. (5) Cell isolation and Culture.
			Neck	HS (36 months)	
3	Male	25	Face	NS	(1) IHC of CILP1 or PPARs protein expression. (2) WB of CILP1 protein expression. (3) RT-qPCR of CILP1 mRNA expression. (4) IF of CILP1 and α-SMA co-staining. (5) Cell isolation and Culture.
			Face	HS (24 months)	
4	Female	54	Hand	NS	(1) IHC of CILP1 or PPARs protein expression. (2) RT-qPCR of CILP1 mRNA expression. (3) IF of CILP1 and α-SMA co-staining. (4) Cell isolation and Culture.
			Hand	HS (12 months)	
5	Female	35	Abdomen	NS	(1) IHC of CILP1 or PPARs protein expression. (2) RT-qPCR of CILP1 mRNA expression. (3) IF of CILP1 and α-SMA co-staining. (4) Cell isolation and Culture.
			Abdomen	HS (18 months)	

*HS* hypertrophic scar, *NS* normal skin, *IHC* immunohistochemistry, *WB* Western blot, *RT-qPCR* quantitative real-time PCR, *IF* Immunofluorescence.

**Table 3:** Characteristics of patients with keloid scar and corresponding normal skin tissues used for immunostaining.

Patient	Sex	Age (years)	Origin	NS or KS (Duration)	Applications
1	Female	32	Earlobe	NS	(1) IHC of CILP1 protein expression. (2) IF of CILP1 and α-SMA co-staining.
			Earlobe	KS (36 months)	
2	Male	26	Chest	NS	(1) IHC of CILP1 protein expression. (2) IF of CILP1 and α-SMA co-staining.
			Chest	KS (24 months)	
3	Female	29	Abdomen	NS	(1) IHC of CILP1 protein expression. (2) IF of CILP1 and α-SMA co-staining.
			Abdomen	KS (12 months)	
4	Female	20	Earlobe	NS	(1) IHC of CILP1 protein expression. (2) IF of CILP1 and α-SMA co-staining.
			Earlobe	KS (48 months)	
5	Female	37	Chest	NS	(1) IHC of CILP1 protein expression. (2) IF of CILP1 and α-SMA co-staining.
			Chest	KS (12 months)	

*HS* hypertrophic scar, *KS* keloid scar, *IHC* immunohistochemistry, *IF* Immunofluorescence.

**Table 4:** Characteristics of patients with hypertrophic scar whose serums were used for ELISA assay.

Patient	Sex	Age (years)	Origin	Duration (months)	Applications
1	Female	12	Chest	8	ELISA
2	Male	32	Neck	10	ELISA
3	Male	17	Cheek	12	ELISA
4	Female	10	Upper arm	6	ELISA
5	Male	33	Cheek	10	ELISA
6	Female	9	Chest	7	ELISA
7	Female	60	Upper arm	12	ELISA
8	Female	22	Upper arm	10	ELISA
9	Female	20	Cheek	12	ELISA
10	Male	55	Hand	8	ELISA
11	Male	29	Cheek	10	ELISA
12	Male	8	Hand	12	ELISA
13	Male	30	Hand	10	ELISA
14	Male	48	Hand	6	ELISA
15	Male	33	Hand	8	ELISA
16	Female	34	Abdomen	8	ELISA
17	Female	19	Head	12	ELISA
18	Female	20	Head	12	ELISA
19	Male	12	Neck	16	ELISA
20	Male	45	Cheek	24	ELISA
21	Female	28	Neck	13	ELISA
22	Female	22	Abdomen	22	ELISA
23	Female	14	Perineum	16	ELISA
24	Male	31	Upper arm	16	ELISA
25	Female	55	Thigh	22	ELISA
26	Female	43	Tempus	60	ELISA
27	Male	14	Head	24	ELISA
28	Male	28	Cheek	48	ELISA
29	Female	12	Chest	24	ELISA
30	Male	32	Chest	24	ELISA
31	Male	22	Abdomen	36	ELISA
32	Male	24	Chest	20	ELISA
33	Female	52	Abdomen	18	ELISA
34	Male	36	Back	16	ELISA
35	Female	21	Shoulder	22	ELISA
36	Female	24	Chest	48	ELISA
37	Female	33	Hand	17	ELISA
38	Male	24	Trunk	20	ELISA
39	Male	19	Upper arm	29	ELISA
40	Male	12	Trunk	16	ELISA
41	Female	16	Forehead	20	ELISA
42	Female	22	Chest	22	ELISA
43	Male	23	Thigh	19	ELISA
44	Male	48	Abdomen	16	ELISA
45	Male	32	Trunk	48	ELISA
46	Female	38	Hand	36	ELISA
47	Female	33	Shoulder	24	ELISA
48	Female	11	Hand	16	ELISA
49	Male	17	Neck	16	ELISA
50	Female	30	Neck	14	ELISA
51	Male	32	Trunk	36	ELISA
52	Female	42	Upper arm	36	ELISA

### Animal model

We obtained the 8-week-old male C57BL/6 mice and New Zealand white rabbits (weight, 3.0–4.0 Kg) from the Experimental Animal Center of the Fourth Military Medical University, China. Animal treatment was conducted in strict accordance with guidelines from the Committee on Publication Ethics. Animal models used for the present study were the load-induced HS mouse model, HS mouse model, and rabbit ear HS model.

The first animal model we used is the load-induced hypertrophic scar mouse model established by Geoffrey C Gurtner [19]. Briefly, the dorsal skin was shaved to remove hairs, later, the skin was treated with the topical depilatory agent for a 30 s period. Firstly, we made a liner incision (2 cm) on dorsal midline in mice, prior to reapproximation using nylon sutures. 4 days later, we removed the sutures, and secured the biomechanical loading device using nylon sutures with caution. On day 4, expansion screws of the device were distracted prudentially by 2 mm to create mechanical load on scars; meanwhile, they were stretched by 4 mm at 2 days intervals later till 2 weeks for maintaining the pressure. Then the scar tissues were obtained and prepared for corresponding assays.

The second animal model is the HS mouse model which was built by Michelle F. Griffin etc [20]. Briefly, the dorsal skin was shaved to remove hairs, then, the skin was treated with the topical depilatory agent for a 30 s duration. Afterwards, a full-thickness excisional wound was made through 6 mm punch biopsy. Then, we put a silicone ring surrounding every wound, and used simple interrupted stitches as well as cyanoacrylate quick drying adhesive to secure the ring for 2 weeks for maintaining the mechanical stretch. Then the scar tissues were collected and prepared for corresponding assays.

The third animal model is the rabbit ear hypertrophic scar model constructed as described in our prior publication [21]. New Zealand white rabbits were intravenously injected with 1% (10 g/L) pentobarbital sodium for anesthesia at 40 mg/Kg. The electric shaver was utilized to remove the rabbit hairs on the ventral side, and later depilatory cream was applied. The 1 cm biopsy punch was used to made 4 full-thickness circular wounds (diameter: 1 cm) on the ventral surface of every ear. The surgical blade was utilized to totally remove the perichondrium within every wound bed out of the cartilage. After one week, crusts were removed for exposing the wounds again. Thirty-five days later, the scar tissues in each group were collected and prepared for corresponding assays.

In the AAV-virus injection experiments, 5 ×10^10^ virus particles with either AAV2-shCILP1 or AAV2-shCtrl were subcutaneously injected in the dorsum of each mouse at 7 day prior to surgery. Totally 12 mice were equally randomly classified into the AAV2-shCtrl group and the AAV2-shCILP1 group. In recombinant human CILP1 protein injection experiments, we administered subcutaneously 100 µL of the solvent or recombinant human CILP1 protein (15 μg/Kg) solution to the wound area of the mice every other day. Totally 12 mice were equally randomized into the control group and the recombinant human CILP1 protein group. On postoperative day 14, the scar tissues on the back of C57BL/6 mice and the surrounding normal skins were collected for H&E, Masson staining and Sirius red staining.

### Transcriptome sequencing and analysis

We utilized Trizol reagent kit (Invitrogen, Carlsbad, CA, USA) for extracting total RNA from HS samples and their corresponding normal skin, whereas Oligo(dT) beads were utilized for total RNA enrichment. Then, fragmentation buffer was added to fragment mRNA into short fragments, and later prepared into cDNA through reverse transcription using random primers. cDNA fragment was purified by QiaQuick PCR extraction kit (Qiagen, Venlo, The Netherlands); later, end repairing, poly(A) addition, and ligation to Illumina sequencing adapters were completed. Agarose gel electrophoresis was conducted to select the size of ligation products, followed by PCR amplification, and sequencing with Illumina Novaseq6000 in Gene Denovo Biotechnology Co. (Guangzhou, China).

### Quantitative real-time PCR (qRT-PCR) assay

RNA simple Total RNA Kit (Invitrogen) was employed to extract total RNAs from obtained tissue and cells. PrimeScript RT Master Mix (Takara, Dalian, China) was adopted to prepare cDNA. In addition, qRT-PCR was performed to measure the target gene levels using SYBR Premix EX Taq (Takara, Dalian, China). The ^ΔΔ^CT approach was utilized to determine the relative target gene mRNA expression, with GAPDH being the endogenous reference. Results were indicated by fold change compared with the control group. Used primers were as follows: Human CILP1: Forward 5’- AGTCTGGAAAGAACATCTCCTGG-3’ and Reverse 5’- CTGGGCTACACTGGGTTTCT-3’; Human PPARα: Forward 5’- ATGGTGGACACGGAAAGCC-3’ and Reverse 5’- CGATGGATTGCGAAATCTCTTGG-3’; Human PPARδ: Forward 5’- CAGGGCTGACTGCAAACGA-3’ and Reverse 5’- CTGCCACAATGTCTCGATGTC-3’; Human PPARγ: Forward 5’- GGGATCAGCTCCGTGGATCT-3’ and Reverse 5’- TGCACTTTGGTACTCTTGAAGTT-3’; Human GAPDH: Forward 5’-ACAACTTTGGTATCGTGGAAGG-3’ and Reverse 5’-GCCATCACGCCACAGTTTC-3’.

### Enzyme-linked immunosorbent assay (ELISA)

Serum CILP1 concentrations in patients with HS or the supernatants of cultured cells were determined with Human CILP1 ELISA kits (CUSABIO, Wuhan, China). The specific steps were conducted in line with corresponding protocols. Absorbance was determined at 450 nm, whereas the CILP1 levels were determined based on the standard curve within the effective range.

### Western blot assay

RIPA lysis buffer supplemented with protease inhibitors was added to homogenize cells and tissues. The protein BCA Protein Assay Kits (Beyotime, Shanghai, China) were adopted for quantifying the protein contents. After separation through sodium dodecyl sulfate–polyacrylamide gel electrophoresis (Beyotime, Shanghai, China), proteins were transferred onto the polyvinylidene difluoride membranes (Millipore, Billerica, USA). Membranes were thereafter blocked for 1 h with 5% nonfat milk at room temperature, prior to overnight incubation using the indicated antibodies under 4 °C. Antibodies utilized in Western blot assay were provided in Antibodies and Reagents section.

### Histological and immunohistochemical staining

After paraformaldehyde (PFA)-fixation overnight, paraffin-embedding and slicing, tissue sections were subjected to hematoxylin and eosin (H&E), Masson’s trichrome or Sirius red staining. In immunohistochemical staining, primary antibodies were added to incubate tissue sections overnight under 4 °C and on the following day, HRP-labeled secondary antibody (ComWin, Beijing, China) was added to incubate sections for 60 min under ambient temperature. The antibodies applied for immunohistochemistry staining were shown in Antibodies and Reagents section. DAB solution (DAKO, Denmark) was added to detect the antibody binding, followed by hematoxylin counterstaining and fixation before microscopic examination.

### Immunofluorescence analysis

For the attached cells, cells were seeded on immunofluorescence dish (801002, Nest) and fixed in 4% PFA for a 15 min and permeabilized in 0.25% Triton X-100 for another 10 min. After 30 min blocking using goat serum under ambient temperature, primary antibodies were added to incubate cells. After cell incubation with appropriate secondary antibodies (Alexa Fluor 488 and 594, Affinity), 4’,6-diamidino-2-phenylindole (DAPI) was introduced for nuclear staining. For the tissue specimens, after deparaffinage of paraffin-embedded sections, the Tris-EDTA buffer (pH 9.0) was added for antigen retrieval. Subsequently, goat serum was introduced to block sections under ambient temperature for a 30 min duration, prior to primary antibody incubation. After further incubation using appropriate secondary antibodies (Alexa Fluor 488 and 594, Affinity), DAPI was added to stain the nuclei. Antibodies utilized for immunofluorescence staining were provided in Antibodies and Reagents section.

### Cell culture

The medium contained 10% fetal bovine serum (BI, Israel) and 1% penicillin-streptomycin. Cells were cultivated with 5% CO_2_ under 37 °C. To isolate human hypertrophic scar fibroblasts (HSFs) and normal fibroblasts (NSFs), phosphate-buffered saline (PBS) that contained 1% penicillin-streptomycin was added to rinse tissues thrice, which were then trimmed to remove the subcutaneous adipose tissue. Subsequently, the rest tissue samples were minced to pieces and placed within culture media. HSFs and NSFs at 3-5 passages were collected for later analyses.

### Transfection of siRNAs and CILP1 overexpression plasmid

The applied CILP1 siRNAs (si-CILP1), YBX1 siRNAs (si-YBX1), TGF-β1 siRNA (si-TGF-β1), PPARα siRNA (si-PPARα), PPARδ siRNA (si-PPARδ), PPARγ siRNA (si-PPARγ), and CILP1 overexpression plasmid was transfected into HSFs according to LipofectamineTM 3000 reagent instructions (Thermo Fisher Scientific, USA). Normal Control siRNA (si-NC), si-CILP1, si-YBX1, si-TGF-β1, si-PPARα, si-PPARδ, and si-PPARγ were constructed by Tsingke Biotechnology Co., Ltd (Beijing, China). siRNA sequences used in this study were shown below: si-CILP1#1: 5’-GGGAUCGAUAUGACUACAA-3’, si-CILP1#2: 5’-CCGUGUUCCAUGAAAUCAA’, si-TGF-β1: 5’-AAGGGCTACCATGCCAACTTC-3’, si-PPARα: 5’- GGAGCAUUGAACAUCGAAU -3’, si-PPARδ: 5’- CCUUCUCCAAGCACAUCUA -3’, si-PPARγ: 5’- GAAGACAUUCCAUUCACAA -3’, siYBX1#1: 5’-GGAGGCAGCAAAUGUUACA-3’, siYBX1#2: 5’-GGAACGGAUAUGGUUUCAU’. CILP1 overexpression plasmid was designed and prepared in Tsingke Biotechnology Co., Ltd (Beijing, China).

### Cell viability assay

Cell Counting Kit-8 assay (Beyotime, China) was carried out to detect the cell viability. In brief, cells were inoculated into 96-well plates and received corresponding treatment. These plates were later subjected to 24 h, 48 h and 72 h incubation under 37 °C separately. At different time points, then culture medium was discarded and replaced using serum-free DMEM (100 μL) that contained 10 μL CCK-8 reagent (Beyotime, China), followed by cell incubation under 37 °C. Two hours later, the medium absorbance was measured at 450 nm at the indicated time points.

### 5-Ethynyl-2’-deoxyuridine (EdU) assay

After inoculation onto the 24-well plates under the indicated treatments, cells were cultured using EdU staining buffer for 4 h, followed by 15-min fixation using 4% PFA under ambient temperature and 15 min permeabilization using PBS that contained 0.3% Triton-X100. After being incubated for 30 min using click reaction solution in dark under ambient temperature, cells were subjected to 10 min staining using DAPI. A fluorescence microscope (Niko, Tokyo, Japan) was employed to examine the stained cells.

### Flow cytometry

We conducted flow cytometry to determine cell cycles. The collected cells were treated with pre-chilled 75% ethanol, followed by 24 h preservation under −20 °C. After discarding ethanol, retained cells were rinsed by PBS under ambient temperature, followed by resuspension within propidium iodide (PI) DNA staining buffer and another 15 min incubation in dark under ambient temperature. The flow cytometer (FACSAria, BD Biosciences) was employed for checking cell cycle.

### Cell migration assay

Cells were inoculated into 6-well plates and received corresponding treatments. Linear defects were created with a pipette tip when the cells reached 90% confluency. The solution containing 2 μg/mL mitomycin C was later added to incubate cells for 2 h. The wells were rinsed thrice using PBS. Defect areas were photographed immediately, 12 h and 24 h after wounding and quantified with ImageJ software.

### Transwell assay

The 24-well inserts (pore size, 8 μm; Corning, NY) were adopted for Transwell assay. Briefly, DMEM that contained 10% FBS (500 μL) was introduced into the bottom chamber, whereas FBS-free DMEM (200 µL) that contained 5000 cells was introduced into the top chamber. Forty-eight hours later, cells migrating into the membrane were fixed prior to crystal violet staining. Images were acquired by microscope and the stained cells were counted for quantification.

### Collagen gel contraction assay

We inoculated cells into 24-well plates that contained collagen suspension (500 μL, Cell Biolabs, San Diego, CA). Then, collagen gel was polymerized, followed by gel release from plates via the slight tiling of plates. Later, collagen gel area was determined immediately, 24 h and 48 h. Then, we employed Image J software for quantification.

### Mass spectrometry (MS)

To conduct protein Co-IP and MS, 5 μg primary CILP1 antibody or IgG (30000-0-AP, Proteintech) was added to incubate approximately 1000 μg total protein overnight, followed by 4 h treatment using Protein A/G PLUS-Agarose (20 μL). The collected immunoprecipitate was subjected to SDS–PAGE for separation. Commercial iST Sample Preparation kit (PreOmics) was used for gel lysis and protein digestion. Then, nano-HPLC–S/MS was conducted to analyze the peptides, whereas PEAKS Studio version 10.6 (Bioinformatics Solutions Inc) was utilized for data processing in Gene Denovo Biotechnology Co.

### Co-immunoprecipitation (Co-IP) assay

IP lysis buffer (P0013, Beyotime) that contained protease/phosphatase inhibitor cocktail was utilized to collect cell lysates as described previously. Then whole-cell lysates (WCLs) were incubated with IP antibody (5 µg) overnight with gentle rocking at 4 °C. Later, we introduced 20 µL protein A/G agarose beads (sc2003, Santa Cruz) for another 4 h incubation. Mixtures were subjected to 5 min centrifugation at 2500 × *g* and 4 °C for bead collection. After careful washing using IP washing buffer containing protease/phosphatase inhibitor cocktail for 5 times, beads were resuspended with 40 μL SDS-PAGE loading buffer prior to additional 10 min boiling. SDS-PAGE and Western blot assay were carried out according to previous description.

### Molecular docking

CILP1 and YBX1 crystal structures were downloaded from Protein Data Bank and were pretreated by the Protein Preparation Wizard module in Schrodinger Software. We used protein-protein docking module to predict protein-protein docking and Protein Interaction Analysis module to determine specific regions of protein binding.

### AAV (adeno-associated virus) vector (AAV2) transfection

The PAAV-CMV was used to generate virus, followed by purification according to prior descriptions. Interference sequences used were shown below: CILP1 shRNA: 5’-GCATGTGCCAGGACTTCATGC-3’. Scrambled shRNA was applied as negative control. The products were synthesized by Tsingke Biotechnology Co., Ltd.

### GST pull-down assay

GST-CILP1 and His-YBX1 fusion proteins were expressed in E. coli and purified. Then, purified GST-CILP1 protein was employed to bind to glutathione agarose and used for binding assays with purified His-YBX1 protein. Briefly, His-YBX1 was allowed to associate with the beads carrying either GST or GST-CILP1 for 3 h in GST pull-down binding buffer (20 mM Tris; 100 mM NaCl; 1 mM EDTA; 1 mM DTT) at 4 °C. The protein complexes were washed four times in the same buffer, dissociated by boiling in loading buffer, and processed with Western blot as described above.

### Nuclear and cytoplasmic protein extraction assay

Nuclear and Cytoplasmic Protein Extraction Kit (P0027, Beyotime) was used to extract nuclear and cytoplasmic protein. In short, cells (20 μL) were collected and resuspended in cytoplasmic protein extraction reagent A (200 μL) and vortexed violently. After addition of cytoplasmic protein extraction reagent B (10 μL), the supernatant (cytoplasmic protein) and sediment was separated by centrifuge. Nuclear protein extraction reagent (50 μL) was used to lyse the sediment and the supernatant (nuclear protein) was obtained using centrifugation. Nuclear and cytoplasmic protein was measured by Western blot assay.

### Chromatin immunoprecipitation (ChIP) assay

Lysates obtained in HSFs were utilized in ChIP assay using SimpleChIP® Enzymatic Chromatin IP Kit SimpleChIP Plus Sonication Chromatin IP Kit (#56383, CST, Danvers, MA, USA) according to the specific protocol. Briefly, primary antibody and isotype control IgG antibody (Antibodies utilized in ChIP were provided in Antibodies and Reagents section) were added for immunoprecipitation. Through phenol–chloroform extraction and ethanol precipitation, we purified immunoprecipitated DNA and starting DNA extraction from cell lysate aliquots after RNase A and proteinase K treatment. qRT-PCR was carried out to examine the purified genomic DNA. We determined % of Input through 100% × 2(^–△CT^), and ^△^CT = Ct (Ip) – Ct (Input). Used primers were as follows: Human PPARα: Forward 5’- GTTTTCTCTCCCTAAAACCTTGGG-3’ and Reverse 5’- ACCTCCGGGCTCAAAGACA-3’; Human PPARδ: Forward 5’- GTGAGGAGCCTCTCCGCCCGG-3’ and Reverse 5’- TGGCCCGTTCTCAATGAGCTGTT-3’; Human PPARγ: Forward 5’- GCTGAGATTACAGGCACGTG-3’ and Reverse 5’- GTATGGCAGCTCACGCCTGTAATC-3’.

### Dual luciferase reporter assay

In brief, 293 T cells (8 × 10^4^/well) were inoculated into the 6-well plates 1 day prior to transfection. pGL3 PPARs (WT/mutsite)-Luc and YBX1 gene overexpression plasmid was designed and prepared in Tsingke Biotechnology Co., Ltd (Beijing, China). They were transfected according to the Lipofectamine^TM^ 3000 reagent instructions (Thermo Fisher Scientific, USA). Afterwards, cell lysis was conducted, followed by measurement of luciferase reporter activities with Luciferase Reporter system (RG005, Beyotime) by normalizing firefly luciferase activity to Renilla luciferase activity.

### Antibodies and reagents

The following antibodies were used for this study: CILP1 (ab192881, Abcam; PAC382Hu01, Cloud-Clone), COL I (14695-1-AP, Proteintech), α-SMA (67735-1-Ig, 14395-1-AP, Proteintech), COL III (22734-1-AP, Proteintech), MMP2 (10373-2-AP, Proteintech), MMP9 (10375-2-AP, Proteintech), VIM (60330-1-Ig, Proteintech), TGF-β1 (ab215715, Abcam), p-Smad2/3 (#8828, Cell Signaling), Smad2/3 (#8685, Cell Signaling), p-ERK1/2 (sc-7383, Santa Cruz), ERK1/2 (sc-514302, Santa Cruz), PPARα (66826-1-Ig, Proteintech), PPARδ (60193-1-Ig, Proteintech; ABclonal, A5656), PPARγ (16643-1-AP, Proteintech), YBX1 (20339-1-AP, Proteintech; sc-101198, Santa Cruz), Flag (20543-1-AP, Proteintech), GAPDH (60004-1-Ig, Proteintech).

Recombinant human TGF-β1 protein (HY-P7118) was obtained from MedChemExpress and reconstituted in PBS. Recombinant human CILP1 protein (5504-CP) was obtained from R&D Systems and reconstituted in PBS. CHX(HY-12320), SB431542 (HY-10431), GW6471 (HY-15372), GSK3787 (HY-15577), GW9662 (HY-16578), and Norathyriol (HY-N1029) were obtained from MedChemExpress and reconstituted in DMSO.

### Statistical analysis

Each assay was conducted thrice. Results were indicated by the mean ± SD and analyzed with Graphpad Prism 9.0. For paired data, Student’s *t* test was performed to compare two groups, while for unpaired data, Student’s *t* test with Welch’s correction was used for comparison. Meanwhile, three or more groups were compared by one-way ANOVA with Bonferroni’s correction. ^*^, ^**^, and ^***^ indicated *p* < 0.05, 0.01, and 0.001 respectively.

## Results

### The CILP1 expression is upregulated in various kinds of skin fibrosis tissues obtained from human and animal models

**Fig. 1:**
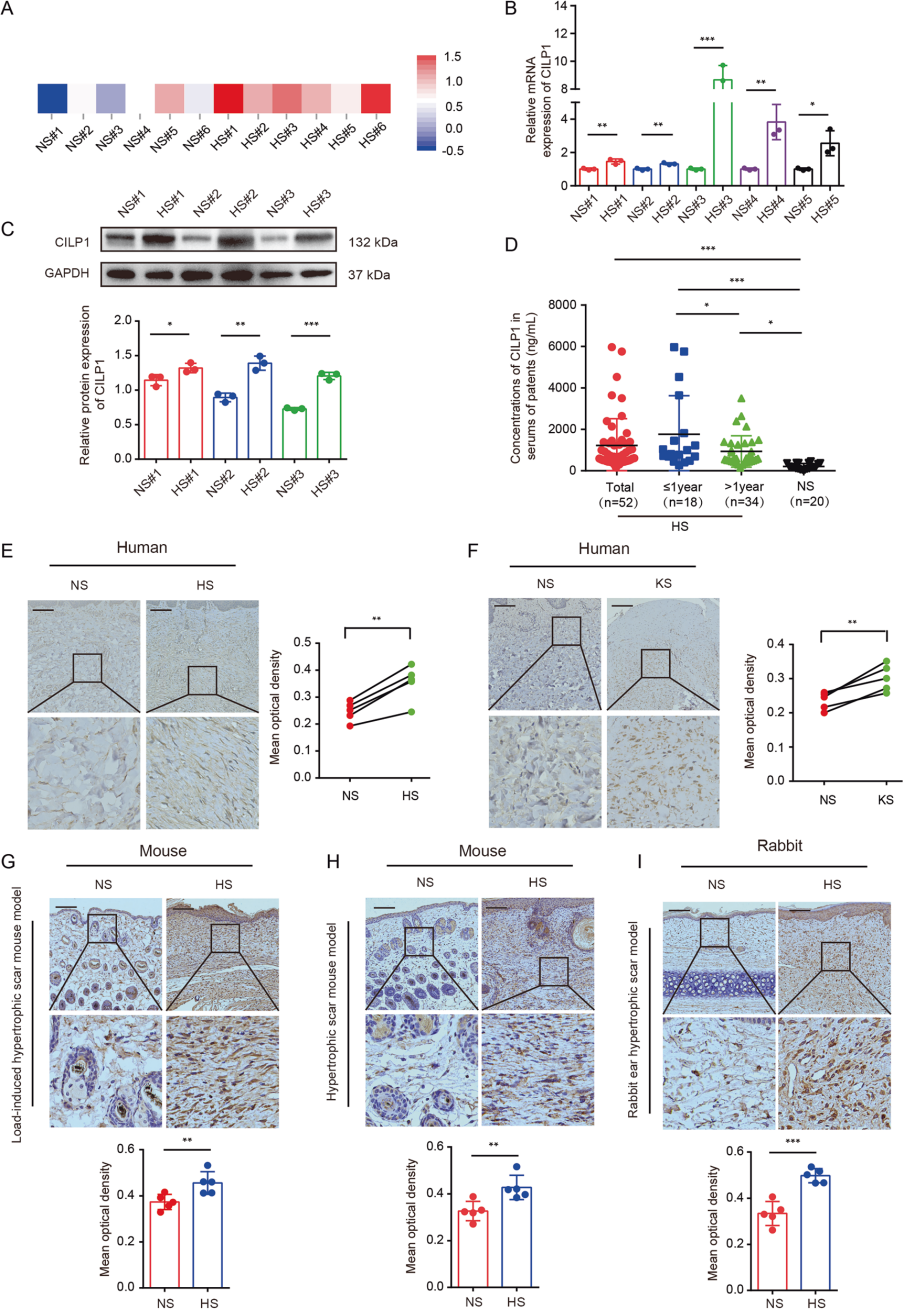
The expression of CILP1 was increased in human skin fibrotic tissues, various animal models of skin fibrosis, and serums of HS patients. **A** RNA sequencing results of CILP1 in six pairs of human NS and HS tissues (*n* = 6). **B** qRT-PCR demonstrated the increased expression of CILP1 in five pairs of NS and HS tissues (*n* = 5). **C** Western blot demonstrated the elevated expression of CILP1 in three pairs of NS and HS tissues (*n* = 3). **D** ELISA revealed the levels of CILP1 in serums of patients with HS within one year (*n* = 18), patients with HS over one year (*n* = 34), and healthy controls (*n* = 20). **E** The results of immunohistochemistry staining for CILP1 in five pairs of human NS and HS tissues (*n* = 5). Scale bar = 100 µm. **F** The immunohistochemistry staining for CILP1 within five pairs of human NS and KS (keloid scar) samples (*n* = 5). Scale bar = 100 µm. **G** Representative images of CILP1 immunohistochemistry staining in the scar tissues of the load-induced hypertrophic scar mouse model (*n* = 5 per group). Scale bar = 100 µm. **H** Representative images of CILP1 immunohistochemistry staining in the scar tissues of the hypertrophic scar mouse model (*n* = 5 per group). Scale bar = 100 µm. **I** Immunohistochemistry staining of CILP1 in scar tissues obtained from the rabbit ear hypertrophic scar model (*n* = 5 per group). Scale bar = 100 µm. Sample size is indicated as individual plots in column graphs. Data are shown as mean ± SD. ^*^P < 0.05, ^**^P < 0.01, ^***^P < 0.001.

To screen the differentially expressed genes (DEGs) in hypertrophic scar, we performed RNA-seq with six pairs of NS and HS tissues, a significantly increased CILP1 expression was observed within HS tissues relative to NS counterparts (Fig. 1A). Based on qRT-PCR and Western blot assays, CILP1 showed increased mRNA and protein expression in HS tissues (Fig. 1B, C). To further explore the potential prediction and diagnostic significance of CILP1 in hypertrophic scar, we collected serum samples from seventy-two individuals, consisting of patients (*n* = 18) with HS diagnosed within one year, patients (*n* = 34) with HS diagnosed for more than one year, and healthy controls (*n* = 20). Then the CILP1 levels were evaluated by ELISA. The results showed that the CILP1 concentrations were significantly higher in all HS patients compared to healthy controls. Notably, among the samples, CILP1 concentrations were highest in patients diagnosed within one year, followed by those diagnosed for more than one year, and were lowest in the healthy controls, with statistically significant differences between these groups (Fig. 1D). Further, to investigate the CILP1 expression characteristics in more skin fibrosis diseases other than hypertrophic scar, immunohistochemical staining was applied, which suggested the major distribution of CILP1 in dermis, and the level of CILP1 was higher in human HS and keloid scar (KS) tissues compared to human NS tissues (Fig. 1E, F). To further verify the elevated expression of CILP1 in skin fibrosis, we established load-induced HS mouse model, HS mouse model, and rabbit ear HS model. IHC analysis verified significant CILP1 protein up-regulation in all the three animal models of skin fibrosis relative to control animals (Fig. 1G–I). These results revealed the increased expression of CILP1 in both human skin fibrotic tissues and skin fibrotic animal models, indicating its potential pro-fibrotic role and clinical significance of hypertrophic scar prediction and treatment.

**Fig. 2:**
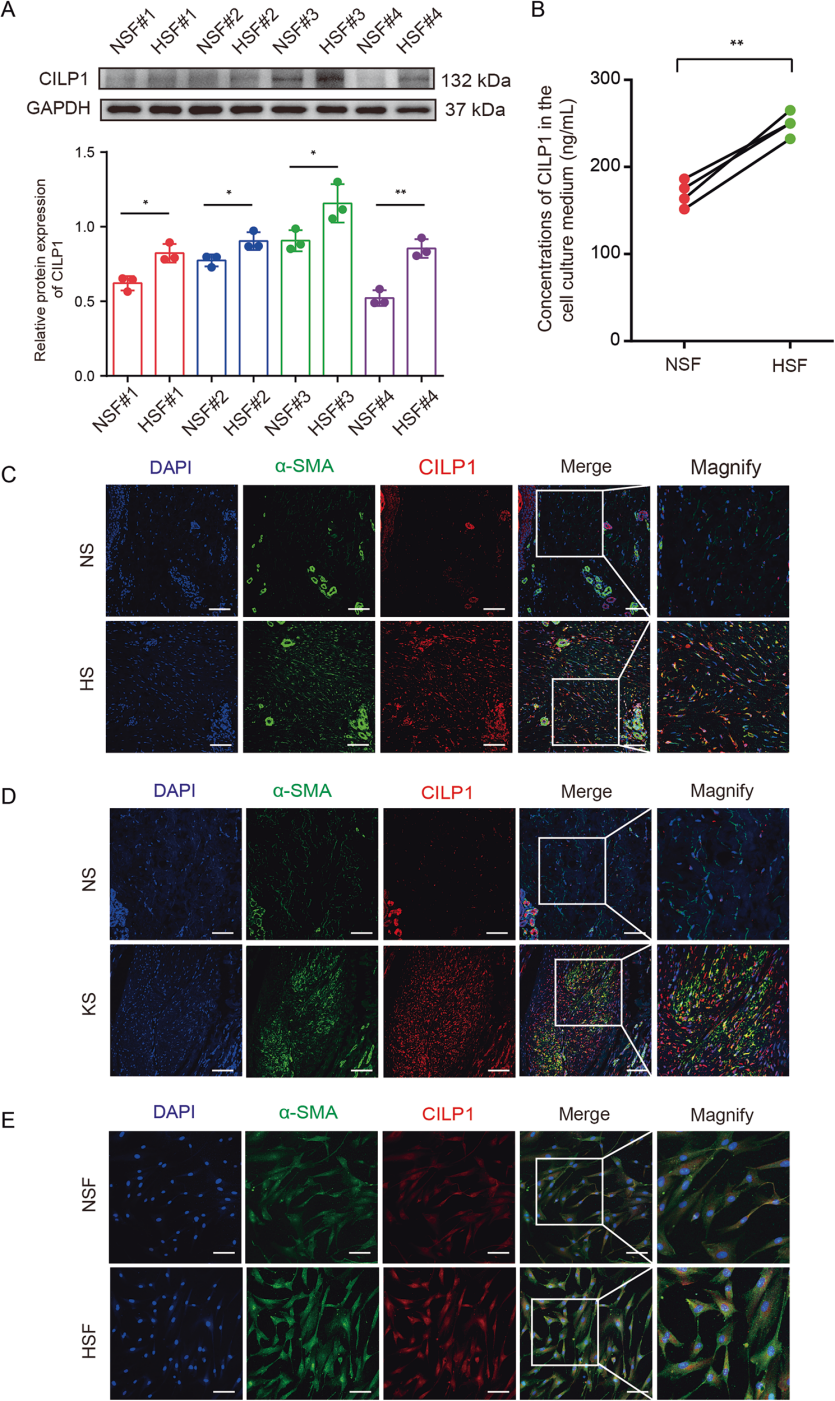
Upregulated levels of CILP1 in HSFs and HSFs cell culture medium. **A** Western blot results of CILP1 in four pairs of NSFs and HSFs (*n* = 4). **B** The results of ELISA demonstrated the increased concentrations of CILP1 in the cell culture supernatants of four pairs of HSFs and NSFs (*n* = 4). **C** Immunofluorescence results showed the co-staining of CILP1 and α-SMA in NS and HS tissues. Scale bar = 100 μm. **D** Immunofluorescence results showed the co-staining of CILP1 and α-SMA in NS and KS tissues. Scale bar = 100 μm. **E** Immunofluorescence results exhibited that CILP1 was co-stained with α-SMA in NSFs and HSFs. Scale bar = 100 µm. Sample size is indicated as individual plots in column graphs. Data are displayed as mean ± SD. ^*^P < 0.05, ^**^P < 0.01.

In addition to fibrotic tissues, we also examined the expression patterns of CILP1 in cultivated primary HS fibroblasts derived from the skin of HS patients and healthy individuals, namely as HSFs and NSFs, respectively. As revealed by Western blot assay, CILP1 expression was markedly higher within HSFs than NSFs (Fig. 2A). Then we carried out ELISA for evaluating the CILP1 levels in culture supernatants of four pairs of HSFs and NSFs, and elevated concentrations of CILP1 were found in the HSFs culture medium, which was similar to the Western blot results (Fig. 2B). In addition, immunofluorescence analysis was employed for revealing the CILP1 and α-SMA co-localization within HS tissues and KS tissues and a large number of cells with CILP1 and α-SMA colocalization were observed (Fig. 2C, D). Moreover, double staining of CILP1 with α-SMA was also conducted to observe the co-localization in human HSFs and NSFs (Fig. 2E). These results further confirmed the increased expression and secretion of CILP1 in HSFs and myofibroblasts, suggesting that CILP1 may function in both the autocrine and paracrine ways and the up-regulation of CILP1 in HSFs may contribute to myofibroblasts differentiation of HS tissues.

### CILP1 contributes to HSFs proliferation and migration

**Fig. 3:**
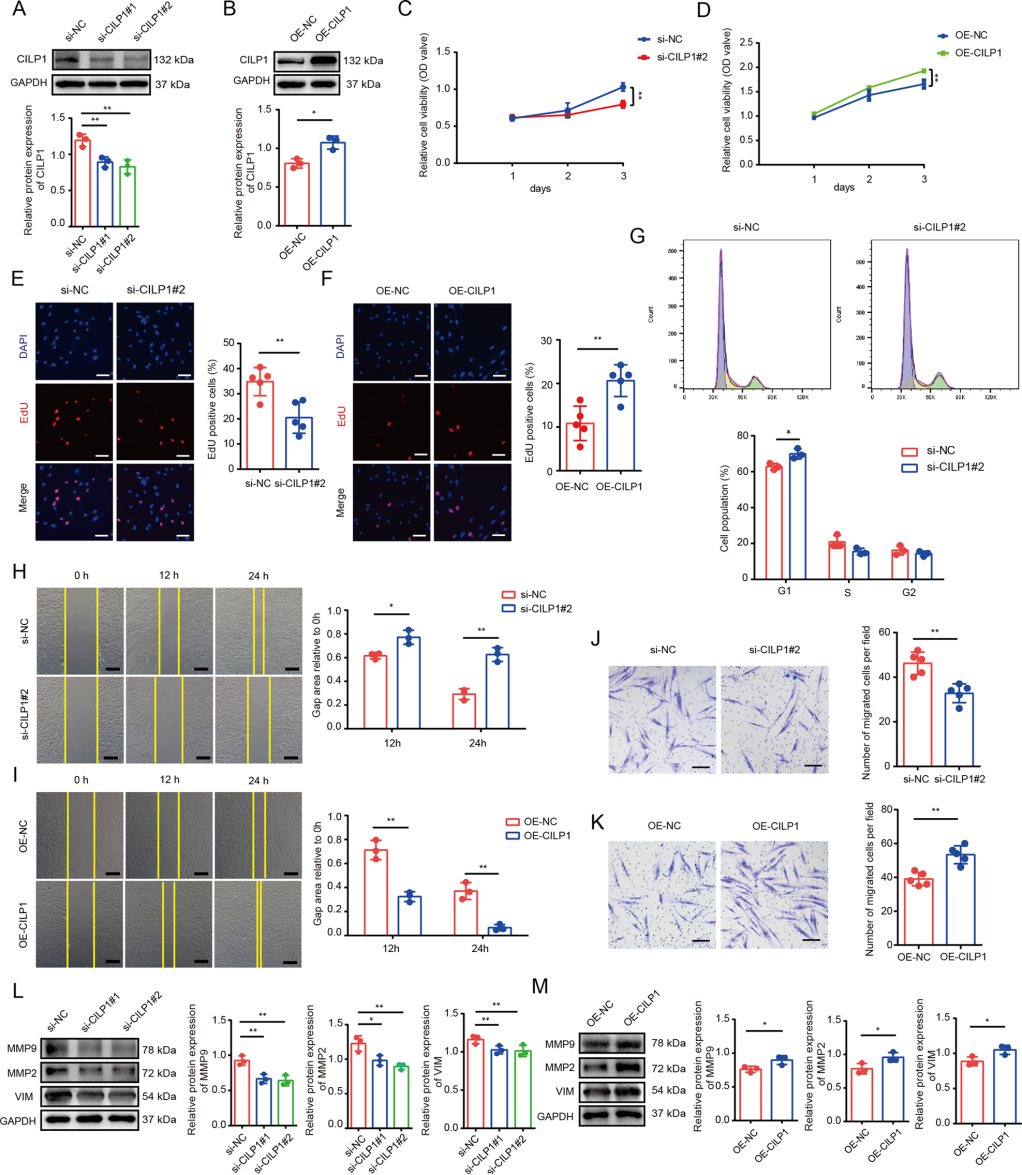
CILP1 contributed to HSFs proliferation and migration. **A** Knockdown efficiency of si-CILP1 in HSFs (*n* = 3). **B** The overexpression efficiency of OE-CILP1 in HSFs (*n* = 3). **C** CCK-8 assay revealed the inhibition of knocking down CILP1 with si-CILP1#2 on HSFs proliferation (*n* = 3). **D** CCK-8 assay demonstrated the enhancement of CILP1 overexpression on HSFs proliferation. **E** EdU staining of HSFs after being treated with si-NC or si-CILP1#2 (Quantitative analysis of EdU-positive cell proportion. *n* = 5 Scale bar = 100 µm). **F** EdU staining of HSFs after being treated with OE-NC or OE-CILP1. (Quantitative analysis of EdU-positive cell proportion. *n* = 5. Scale bar = 100 µm). **G** Flow cytometry results and quantification of cell cycles in HSFs treated with si-NC or si-CILP1#2 (*n* = 3). **H** Images and wound healing assay results of si-NC or si-CILP1 groups (Dotted lines indicate the scratch areas. *n* = 3. Scale bar = 200 µm.). **I** Images and wound healing assay results of OE-NC and OE-CILP1 groups (Dotted lines indicate the scratch areas. *n* = 3. Scale bar = 200 µm). **J** Images and Transwell assay results of si-NC and si-CILP1 groups (*n* = 3. Scale bar = 100 µm). **K** Images and Transwell assay results of OE-NC and OE-CILP1 groups (*n* = 3. Scale bar = 100 µm). **L** MMP2, MMP9, and VIM levels in HSFs after si-NC or si-CILP1 treatment through Western blot assay (*n* = 3). **M** Western blot analysis on MMP2, MMP9, and VIM levels in HSFs after OE-NC and OE-CILP1 treatment (*n* = 3). Sample size is indicated as individual plots in column graphs. Data are presented as mean ± SD. ^*^P < 0.05, ^**^P < 0.01, ^***^P < 0.001.

For exploring the impact of CILP1 expression on HSFs, two independent siRNAs against CILP1 (si-CILP1#1 and si-CILP1#2) were used to knock down the mRNA expression of CILP1, and si-CILP1#2 displayed a higher knockdown efficiency and was chosen for further study (Fig. 3A). In addition, the overexpression plasmid of CILP1 was constructed and applied to up-regulate the expression of CILP1 in HSFs (Fig. 3B). The results of Cell Counting Kit-8 (CCK-8) assay revealed a decreased proliferation of HSFs after CILP1 knockdown (Fig. 3C), while overexpression of CILP1 significantly enhanced HSFs proliferation (Fig. 3D). The result was further confirmed by 5-ethynyl-2’-deoxyuridine (EdU) staining in HSFs upon CILP1 knockdown and overexpression (Fig. 3E, F). Flow cytometry showed substantial G1 phase arrest in HSFs suffered from CILP1 knockdown (Fig. 3G). Further, CILP1 knockdown decreased the vertical and horizontal migration of HSFs (Fig. 3H, J), while CILP1 overexpression significantly augmented HSFs migration (Fig. 3I, K). In addition, knocking down CILP1 reduced MMP2, MMP9, and VIM protein expression, while CILP1 overexpression up-regulated the expression of those genes (Fig. 3L, M). To sum up, these results demonstrated the promotive effects of CILP1 on HSFs proliferation and migration.

### CILP1 promoted fibroblast activation

**Fig. 4:**
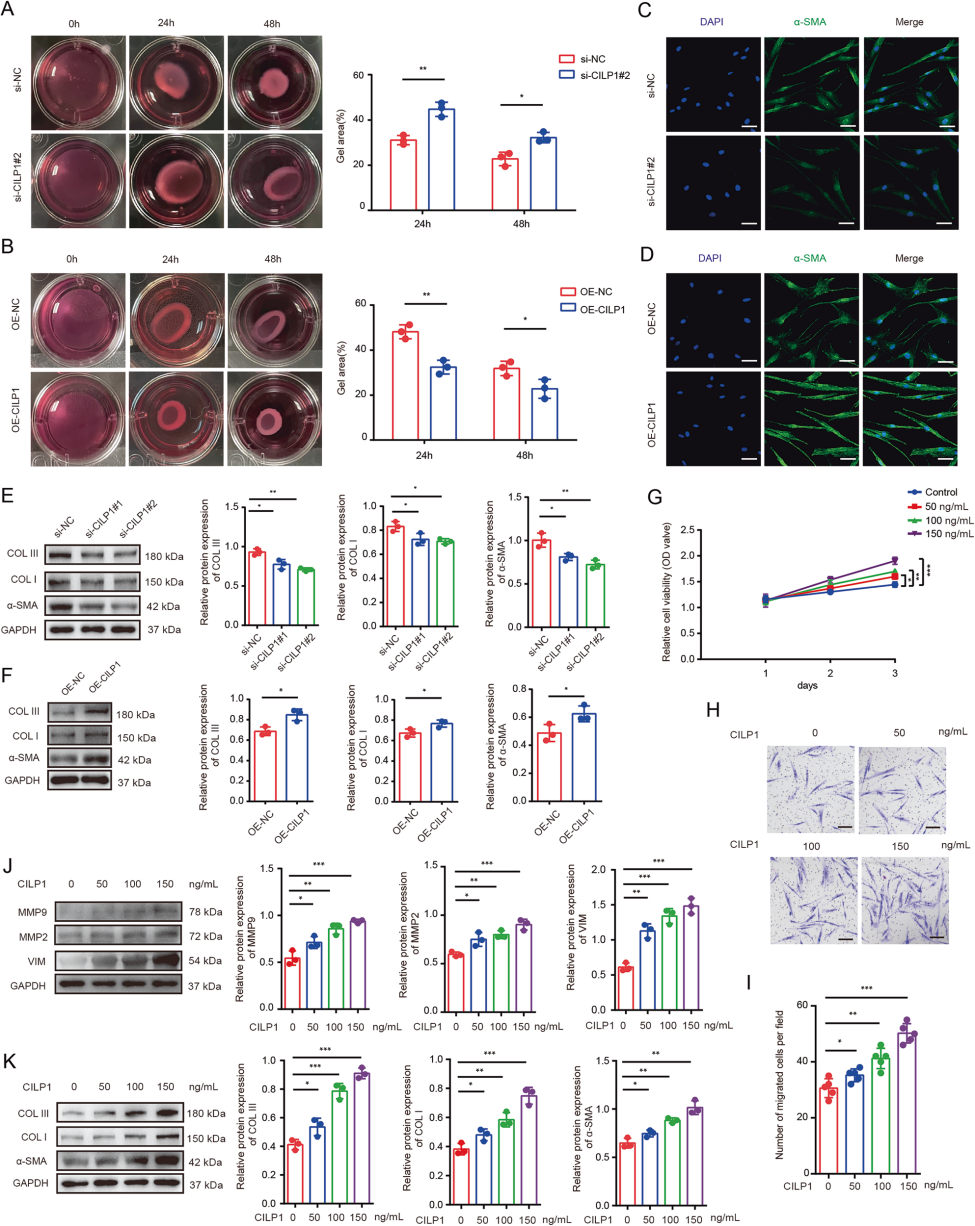
CILP1 promoted cell activation, proliferation, migration, and extracellular matrix synthesis of HSFs. **A** Images and collagen gel contraction quantification results of si-NC and si-CILP1 groups (*n* = 3). **B** Images and collagen gel contraction quantification assays of OE-NC and OE-CILP1 groups (*n* = 3). **C** Immunofluorescence showed the α-SMA staining results in HSFs treated with si-NC or si-CILP1#2. Scale bar = 100 µm. **D** Immunofluorescence showed the α-SMA staining results in HSFs treated with OE-NC or OE-CILP1. Scale bar = 100 µm. **E** Western blot assay showed α-SMA, COL I, and COL III levels in HSFs treated with si-NC or si-CILP1#2 (*n* = 3). **F** Results of Western blot exhibited α-SMA, COL I, and COL III levels within HSFs treated with OE-NC and OE-CILP1 (*n* = 3). **G** CCK-8 assay showed the proliferative capacity of HSFs treated with recombinant human CILP1 protein (0, 50, 100, 150 ng/mL) (*n* = 3). **H**, **I** Images and quantitative results of the Transwell assay in HSFs treated with recombinant human CILP1 protein (0, 50, 100, 150 ng/mL) (*n* = 5). **J** Western blot assay detected MMP2, MMP9, and VIM levels in HSFs treated with recombinant human CILP1 protein (0, 50, 100, 150 ng/mL) (*n* = 3). **K** Results of Western blot showed α-SMA, COL I, and COL III levels in HSFs treated with recombinant human CILP1 protein (0, 50, 100, 150 ng/mL) (*n* = 3). Sample size is indicated as individual plots in column graphs. Results are indicated by mean ± SD. ^*^P < 0.05, ^**^P < 0.01, ^***^P < 0.001.

Then, we analyzed the effects of CILP1 on cell contractive ability of HSFs by collagen gel contraction assay. The data showed that CILP1 knockdown in HSFs reduced collagen gel contraction (Fig. 4A), while CILP1 overexpression enhanced HSFs cell contractive ability (Fig. 4B). Immunofluorescence staining revealed that α-SMA was down-regulated within si-CILP1 HSFs and up-regulated in OE-CILP1 HSFs (Fig. 4C, D). According to Western blot assays, CILP1 knockdown reduced the α-SMA, COL I, and COL III levels (Fig. 4E). CILP1 overexpression group showed markedly elevated α-SMA, COL I, and COL III protein expression relative to the control group (Fig. 4F). Considering that CILP1 can be secreted from HSFs and detected in the cell culture medium of HSFs, which implied that CILP1 may also serve as an extracellular stimulating factor to impact on HSFs in both autocrine and paracrine ways, thus we stimulated HSFs with the recombinant human CILP1 protein and found that recombinant human CILP1 protein can promote HSFs activation, proliferation, migration, and extracellular matrix synthesis (Fig. 4G–J). Further, to explore the role of intracellular CILP1 in promoting fibrosis, we added CILP1 neutralizing antibodies into the conditioned medium to block the influences of extracellular CILP1. Western blot results showed that CILP1 knockdown reduced the expression of fibrosis-associated protein comprising COL I, COL III, and α-SMA and migration-associated protein expression comprising MMP2, MMP9, and VIM (Supplementary Fig. 1A and C), while CILP1 overexpression increased the expressions of these proteins (Supplementary Fig. 1B and D). These confirmed that both the extracellular and intracellular CILP1 harbors the function to facilitate hypertrophic scar formation.

### CILP1 and TGF-β pathway formed a negative feedback loop in HSFs

**Fig. 5:**
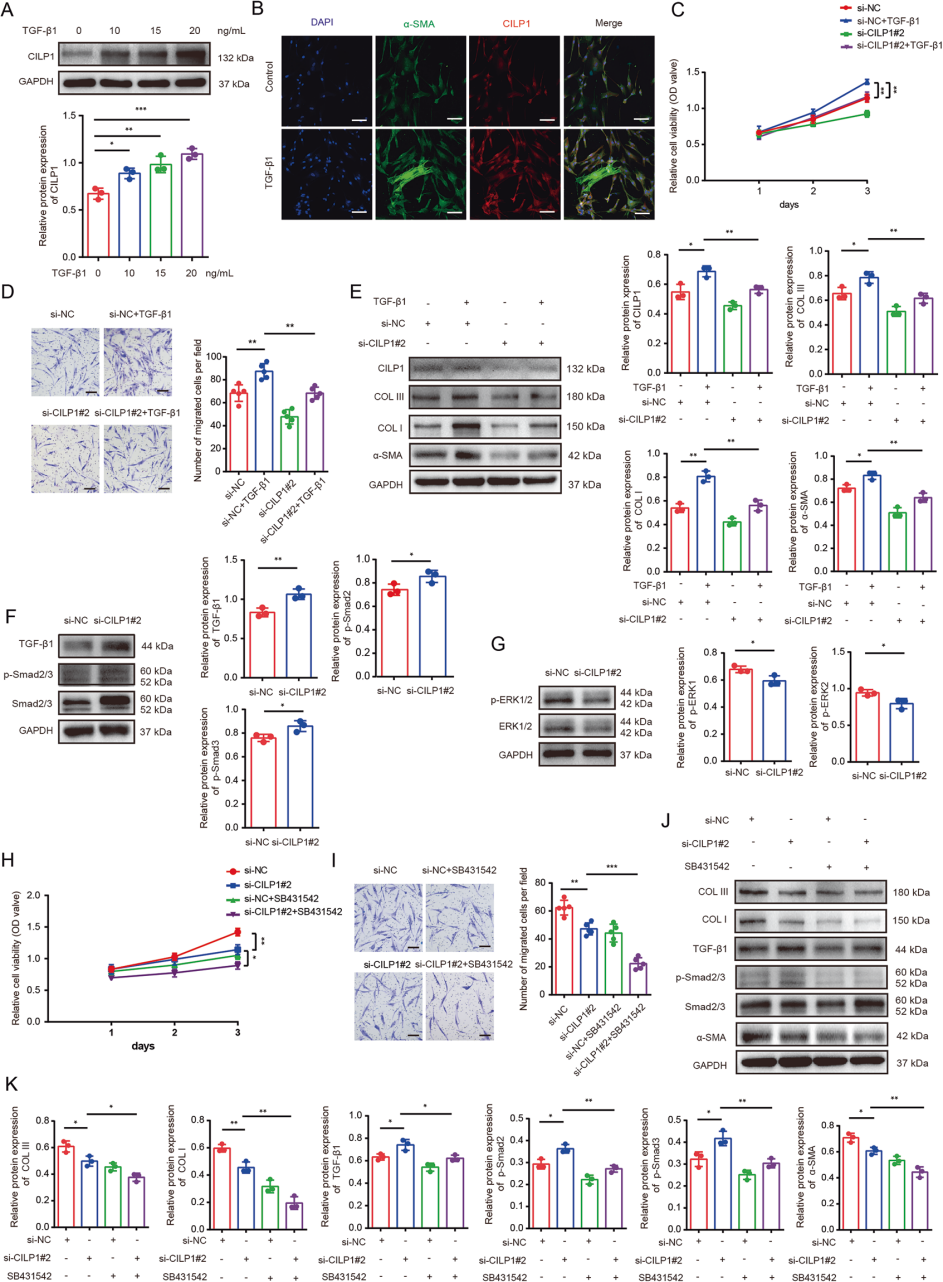
CILP1 and TGF-β pathway formed a negative feedback loop in HSFs. **A** Western blot assay showed CILP1 level in HSFs under stimulation with TGF-β1 (0, 10, 15, 20 ng/mL) for 48 h (*n* = 3). **B** Results of immunofluorescence displayed the expression of CILP1 and α-SMA expression in HSFs after TGF-β1 treatment (0, 10 ng/mL). **C** CCK-8 assay exhibited the proliferative capacity of HSFs after knocking down CILP1 with si-CILP1#2 or/and stimulation with TGF-β1 (0, 10 ng/mL) (*n* = 3). **D** Images and Transwell assay results of HSFs following knocking down CILP1 with si-CILP1#2 or/and stimulation with TGF-β1 (0, 10 ng/mL) (*n* = 5). **E** CILP1, α-SMA, COL I, and COL III protein expression in HSFs after knocking down CILP1 or/and stimulation with TGF-β1 (0, 10 ng/mL) (*n* = 3). **F** Western blot results showed that knocking down CILP1 with si-CILP1#2 activated TGF-β pathway in HSFs (*n* = 3). **G** Western blot results displayed that knocking down CILP1 with si-CILP1#2 significantly inhibited ERK1/2 pathway in HSFs (*n* = 3). **H** CCK-8 assay showed the proliferative capacity of HSFs after knocking down CILP1 with si-CILP1#2 or/and stimulation with TGF-β1 pathway inhibitor SB431542 (0, 10 μM) (*n* = 3). **I** Images and quantitative results of the Transwell assay of HSFs after knocking down CILP1 with si-CILP1#2 or/and stimulation with TGF-β1 pathway inhibitor SB431542 (0, 10 μM) (*n* = 5). **J**, **K** α-SMA, p-Smad2/3, TGF-β1, COL I, and COL III protein expression in HSFs following indicated treatment (*n* = 3). Sample size is indicated as individual plots in column graphs. Results are indicated by mean ± SD. ^*^P < 0.05, ^**^P < 0.01, ^***^P < 0.001.

Since TGF-β1-treated fibroblast is a well-established and well-accepted cell model to study fibroblast activation in vitro. Then we analyzed whether TGF-β1 affected CILP1 and found that recombinant human TGF-β1 protein up-regulated CILP1expression in HSFs in a concentration-dependent manner (Fig. 5A). ELISA assay results showed that recombinant human TGF-β1 protein stimulation enhanced CILP1 secretion in a concentration-dependent manner (Supplementary Fig. 2A). Knocking down TGF-β1 using si-TGF-β1 could decrease the amount of both intracellular and extracellular CILP1, but cannot totally abate the expression of CILP1 (Supplementary Fig. 3A and B). Further, we explored the influence of TGF-β1 on the stability of the CILP1 protein, then the protein levels of CILP1 in HSFs was detected under TGF-β1 stimulation combined with protein synthesis inhibitor cycloheximide (CHX). The results showed that TGF-β1 stimulation elevated CILP1 protein by enhancing CILP1 protein stability (Supplementary Fig. 2B). Immunofluorescence staining revealed that TGF-β1 could stimulate α-SMA expression in HSFs (Fig. 5B), which suggested that CILP1 may play a role in fibroblast-to-myofibroblast transition through TGF-β pathway. Further studies revealed that knocking down CILP1 with si-CILP1#2 could attenuate the pro-proliferative and the pro-migrative effects and ECM synthesis function of TGF-β1 on HSFs respectively (Fig. 5C–E). Further, both si-TGF-β1 and CILP1 overexpression plasmid OE-CILP1 were applied, the results showed that CILP1 overexpression could rescue the downregulation of α-SMA, COL I and COL III induced by si-TGF-β1 to a large extent (Supplementary Fig. 3C). Interestingly, we also revealed that CILP1 knockdown elevated TGF-β1 and p-Smad2/3 levels and reduced p-ERK1/2 expression in HSFs (Fig. 5F, G). Treating HSFs with TGF-β1 specific inhibitor (SB431542) enhanced the inhibitory effect of si-CILP1#2 on cell proliferation, migration, and ECM synthesis (Fig. 5H–J). These indicated that TGF-β1 served as an upstream regulator of CILP1, and CILP1 and TGF-β pathway formed a negative feedback loop in HSFs.

### CILP1 inhibited PPARs pathway in HSFs

**Fig. 6:**
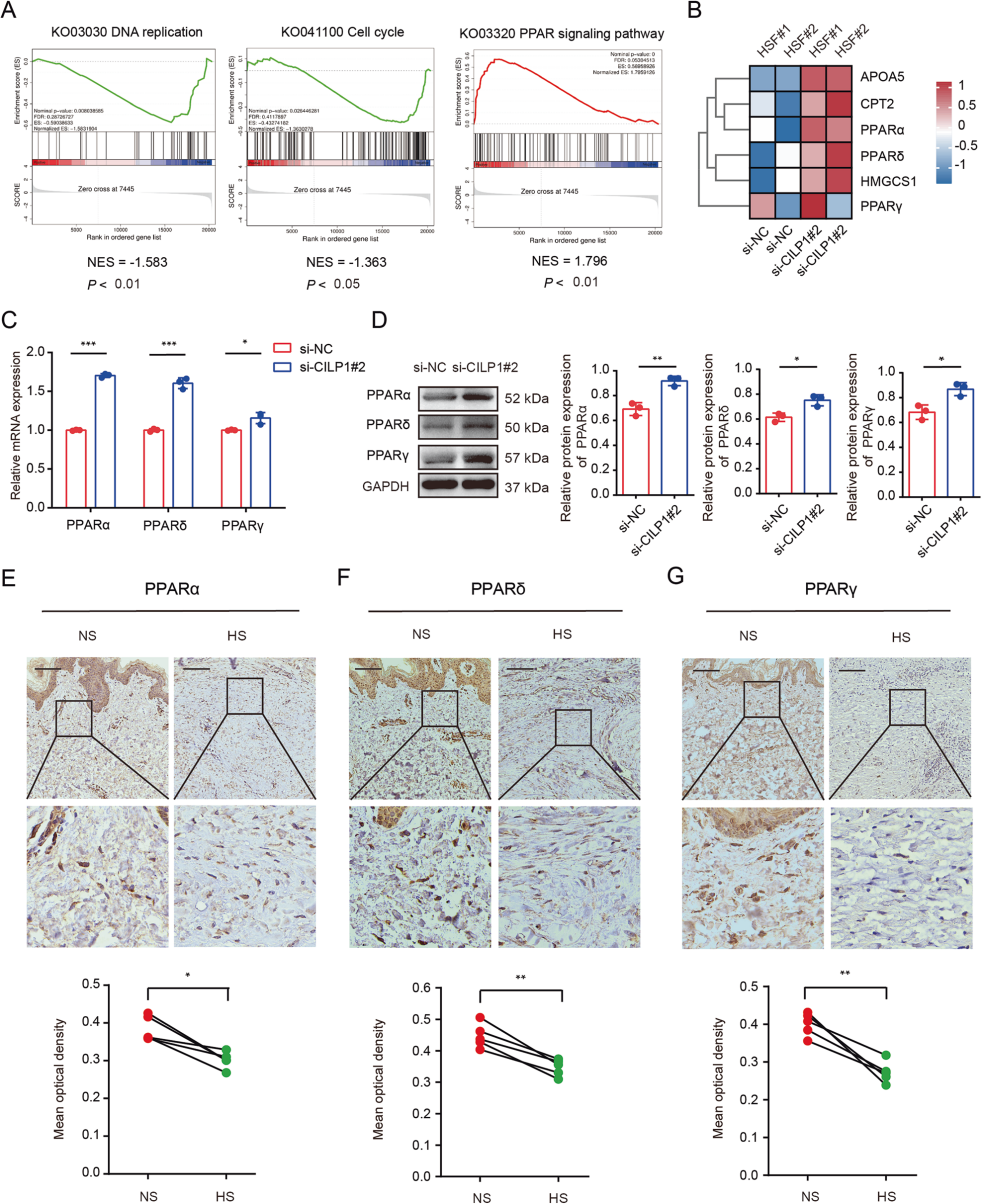
CILP1 suppressed PPARs expression in HSFs and HS tissues. **A** GSEA analysis of differentially-expressed genes (DEGs) after CILP1 knockdown in HSFs. Pathways including PPARs signaling pathway, DNA replication, and Cell cycle are displayed. **B** Heatmap of representative genes significantly regulated by CILP1 in HSFs by RNA sequencing analysis. **C** qRT-PCR results showed that CILP1 knockdown significantly elevated PPARα, PPARδ, and PPARγ mRNA expression in HSFs (*n* = 3). **D** Western blot results showed that CILP1 knockdown significantly increased PPARα, PPARδ, and PPARγ protein expression in HSFs (*n* = 3). **E**–**G** Immunohistochemistry staining exhibited the decreased protein expression of PPARα, PPARδ, and PPARγ in HS relative to their corresponding normal skin in five pairs of human HS and NS tissues (*n* = 5). Scale bar = 100 µm. Sample size is indicated as individual plots in column graphs. Results are indicated by mean ± SD. ^*^P < 0.05, ^**^P < 0.01, ^***^P < 0.001.

**Fig. 7:**
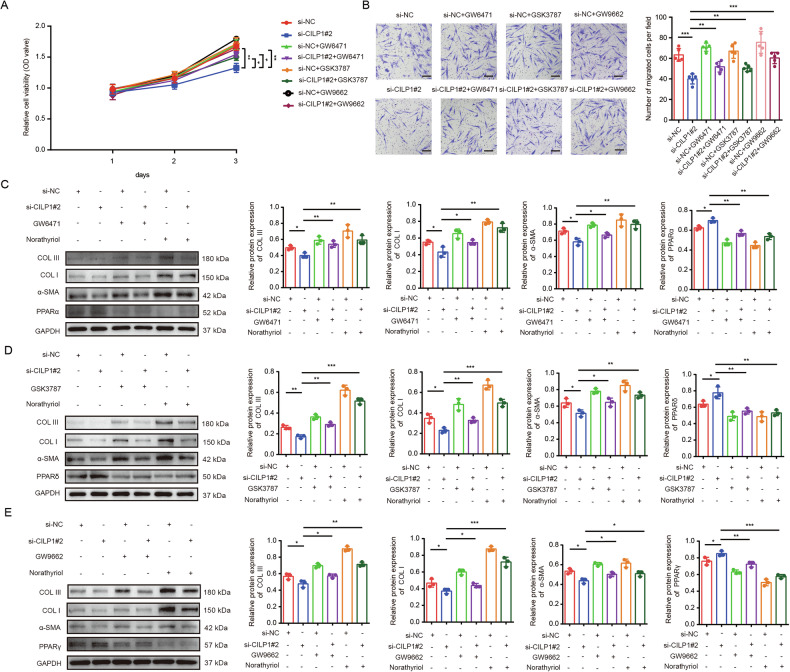
Inhibiting PPARs pathway enhanced the profibrotic effects of CILP1 on HSFs. **A** CCK-8 assay showed the HSFs proliferation after being treated with si-CILP1#2 and/or PPARα inhibitor GW6471 (0, 25 μM), PPARδ inhibitor GSK3787 (0, 5 μM), or PPARγ inhibitor GW9662 (0, 20 μM) (*n* = 3). **B** Images and Transwell assay results of HSFs following indicated treatments (*n* = 5). **C** PPARα, α-SMA, COL I, and COL III protein expression in HSFs after being treated with si-CILP1#2, PPARα inhibitor GW6471 (0, 25 μM), or PPARs pathway pan-inhibitor Norathyriol (0, 25 μM) detected by Western blot (*n* = 3). **D** PPARδ, α-SMA, COL I and COL III protein expression within HSFs after being treated with si-CILP1#2, PPARδ inhibitor GSK3787 (0, 5 μM), or PPARs pathway pan-inhibitor Norathyriol (0, 25 μM) detected by Western blot (*n* = 3). **E** PPARγ, α-SMA, COL I and COL III protein expression in HSFs after being treated with si-CILP1#2, PPARγ inhibitor GW9662 (0, 20 μM), or PPARs pathway pan-inhibitor Norathyriol (0, 25 μM) measured through Western blot (*n* = 3). Sample size is indicated as individual plots in column graphs. Results are indicated by mean ± SD. ^*^P < 0.05, ^**^P < 0.01, ^***^P < 0.001.

Next, we performed RNA-seq for identifying downstream genes of CILP1. As revealed by gene set enrichment analysis (GSEA), DEGs in si-CILP1 versus si-NC groups were mainly associated with several biological pathways, such as PPARs signaling pathway, DNA replication, and Cell cycle, etc (Fig. 6A). Specifically, CILP1 knockdown significantly increased the expression of APOA5, CPT2, PPARα, PPARδ, HMGCS1 and PPARγ, which were implicated in the PPARs signaling pathway (Fig. 6B). Based on the qRT-PCR and Western blot results, knocking down CILP1 up-regulated PPARα, PPARδ, and PPARγ mRNA and protein levels in HSFs (Fig. 6C, D). What’s more, CILP1 overexpression attenuated the protein expressions of PPARα, PPARδ, and PPARγ in HSFs (Supplementary Fig. 4). Adding recombinant human CILP1 protein to the culture medium did not significantly change the expression of PPARα, PPARδ, and PPARγ in HSFs (Supplementary Fig. 5). This indicated that the intracellular CILP1 mainly functions to regulate PPARs synthesis in HSFs. Then we investigated PPARs expression within human NS and HS tissue samples, and decreased protein expression of PPARα, PPARδ, and PPARγ were detected in human HS tissues (Fig. 6E–G). These suggested that CILP1 may facilitate hypertrophic scar formation through inhibiting PPAR pathway. Then the specific inhibitors of PPARα (GW6471), PPARδ (GSK3787), and PPARγ (GW9662) were used to stimulate the HSFs and the results showed that each inhibitor against PPARα, PPARδ, and PPARγ reversed the inhibitory effects of si-CILP1#2 on HSFs proliferation, migration, and ECM synthesis (Fig. 7A–E). Knocking down both CILP1 and PPARs rescued the decreased expression of COL I, COL III, and α-SMA stimulated by CILP1 knockdown (Supplementary Fig. 6). In addition, we also found that PPAR pathway pan-inhibitor Norathyriol which simultaneously inhibited PPARα, PPARδ, and PPARγ exhibited a better effect on reversing the inhibitory function of si-CILP1#2 on HSFs ECM synthesis than individual inhibitors (Fig. 7C–E).

### CILP1 inhibited PPARs pathway in HSFs by interacting with YBX1

**Fig. 8:**
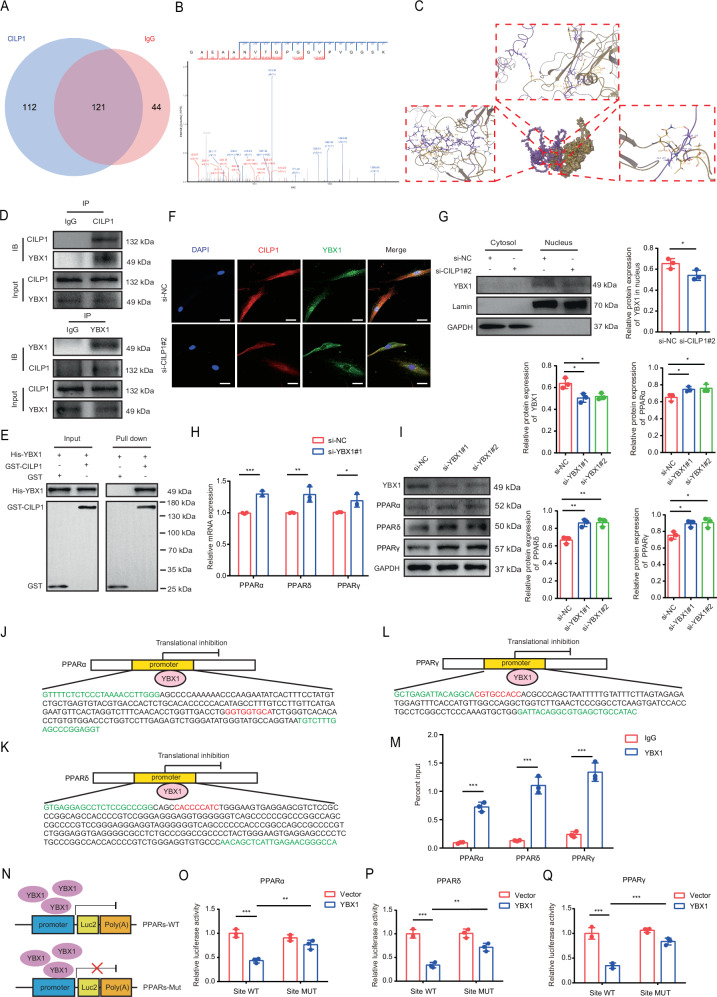
CILP1 suppressed PPARs pathway in HSFs by interacting with YBX1. **A** Venn diagram of CILP1 binding proteins screened by mass spectrometry. **B** Candidate interacting protein of YBX1 identified by mass spectrometry. **C** Molecular docking between CILP1 and YBX1. **D** Co-IP results of CILP1 and YBX1 in HSFs. **E** GST pull-down assay was performed to observe the direct interaction between CILP1 and YBX1. **F** Immunofluorescence staining demonstrated that CILP1 knockdown reduced the expression and nuclear location of YBX1 in HSFs. Scale bar = 50 µm. **G** Nuclear and cytoplasmic protein extraction assay results demonstrated that CILP1 knockdown reduced the YBX1 level in the nuclear fraction of HSFs (*n* = 3). **H** qRT-PCR results showed that YBX1 knockdown significantly elevated PPARα, PPARδ, and PPARγ mRNA expression in HSFs (*n* = 3). **I** Western blot results demonstrated that YBX1 knockdown significantly increased PPARα, PPARδ, and PPARγ protein expression of HSFs (*n* = 3). **J**–**L** JASPAR software predicted the binding sites of YBX1 in the promoters of PPARα, PPARδ, and PPARγ respectively. **M** ChIP-qPCR confirmed the binding of YBX1 to the promoters of PPARα, PPARδ, and PPARγ (*n* = 3). **N**–**Q** The luciferase reporter assay results demonstrated that YBX1 could target these three sites of PPARα, PPARδ, and PPARγ promoters in 293T cells (*n* = 3). Sample size is indicated as individual plots in column graphs. Results are indicated by mean ± SD. ^*^P < 0.05, ^**^P < 0.01, ^***^P < 0.001.

As CILP1 was confirmed to exacerbate HS phenotype through suppressing PPARα, PPARδ, and PPARγ expression, we then explored its underlying mechanism. Pull-down assay plus mass spectrometry (pull-down-MS) was performed to reveal the protein interactome, which obtained 112 potential CILP1-binding proteins within HSFs. Among those candidate proteins, transcription factor Y-box-binding protein 1 (YBX1) was outstanding (Fig. 8A, B) for its powerful pro-fibrotic function. YBX1 is the RNA- and DNA-binding protein that is related to many cellular processes such as regulating translation and transcription, and abnormal expression of YBX1 is linked to fibrosis development in distinct tissues [22–24]. To verify the direct association of CILP1 with YBX1, molecular docking was conducted, which found that CILP1 bound to YBX1 at multiple sites via hydrogen bonds and salt bridges. These two proteins could form a stable complex with the lowest PIPER pose energy (-431.702 kcal/mol) and can be considered to be tightly bound (Fig. 8C). Then, Co-IP was carried out and confirmed the association of CILP1 with YBX1 (Fig. 8D). To verify the direct interaction between CILP1 and YBX1 in vitro, GST pull-down assay was performed, the results confirmed the direct interaction between CILP1 and YBX1 (Fig. 8E). Besides, immunofluorescence analysis demonstrated that CILP1 was co-localized with YBX1 in the nucleus and cytoplasm of HSFs, and CILP1 knockdown reduced the nuclear transport of YBX1 (Fig. 8F). Nuclear and cytoplasmic protein extraction assay further proved that CILP1 knockdown reduced YBX1 nuclear translocation in HSFs (Fig. 8G). We used the Nucleolar localization sequence detector (www.compbio.dundee.ac.uk/www-nod/index.jsp) to identify the nuclear localization sequence (NLS) of CILP1 (TVQGRVPSRRQQRASRGGQRQS) and constructed the plasmid with the mutated bases in the nuclear localization sequence of CILP1. Further immunofluorescence staining and Western blot assays revealed that the wildtype (WT) CILP1 preferred to locate in the nucleus and YBX1 has a tendency to transfer to the nucleus in WT cells, while the mutated (MUT) CILP1 mainly located in the cytoplasm and had no effects on promoting YBX1 nuclear translocation (Supplementary Fig. 7A–C). In addition, the reduced nuclear translocation of CILP1 and YBX1 attenuated the fibrotic phenotype of the HSFs (Supplementary Fig. 7D). Based on these described above, we supposed that CILP1 plays an important role in promoting YBX1 nuclear import and the NLS is functional. Collectively, CILP1 is the transporter for mediating the nuclear transport of YBX1 in HSFs through interacting with YBX1.

Next, we investigated whether the nuclear located YBX1 could transcriptionally regulate PPARα, PPARδ, and PPARγ expression. qRT-PCR and Western blot experiments demonstrated that YBX1 knockdown up-regulated the expression of PPARα, PPARδ, and PPARγ (Fig. 8H, I). Using JASPAR software, we revealed the binding motifs of YBX1 within the promoters of PPARα, PPARδ, and PPARγ respectively (Fig. 8J–L). As verified by ChIP with qPCR (ChIP-qPCR), YBX1 indeed bound to the promoters of PPARα, PPARδ, and PPARγ (Fig. 8M). To further verified that YBX1 directly bound to the promoters of PPARα, PPARδ, and PPARγ rather than through interaction with newly synthesized RNA, we pretreated cell samples with RNase A and performed ChIP-qPCR assays again, the results were in agreement with our previous findings (Supplementary Fig. 8). The luciferase reporter assay further demonstrated that mutating the binding motifs in the promoters of PPARα, PPARδ, and PPARγ impaired the binding of YBX1 to the PPARα, PPARδ, and PPARγ promoters respectively (Fig. 8N–Q). In conclusion, YBX1 transcriptionally modulated PPARα, PPARδ, and PPARγ levels within HSFs. In addition, knocking down YBX1 resulted in the decreased expressions of COL I, COL III, and α-SMA accompanied by the elevation of PPARs comprising PPARα, PPARδ, and PPARγ, knocking down PPARα, PPARδ, or PPARγ increased the expressions of COL I, COL III, and α-SMA, and knocking down both YBX1 and PPARs reversed the YBX1-interference-induced COL I, COL III, and α-SMA downregulation (Supplementary Fig. 9). These confirmed that CILP1 promotes hypertrophic scar formation via the YBX1-PPARs axis.

### CILP1 knockdown mitigated HS formation in vivo

**Fig. 9:**
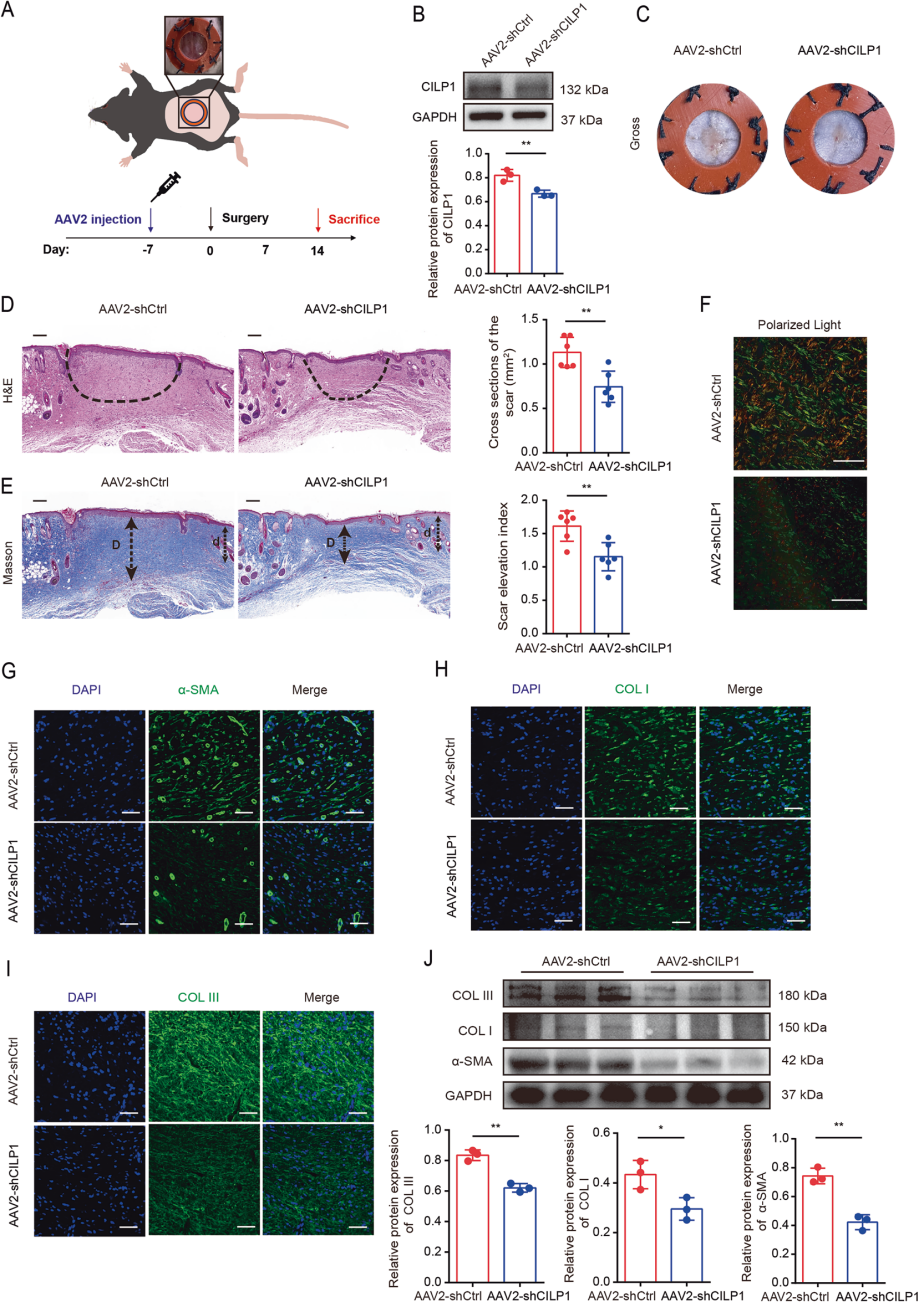
CILP1 knockdown attenuates hypertrophic scar formation in C57BL/6 mice. **A** Schematic diagram of mice hypertrophic scar model construction. **B** Validation of AAV2-mediated knockdown efficiency of CILP1 in mice using Western blot assay (*n* = 3). **C** Representative gross photographs of scars of mice from AAV2-shNC and AAV2-shCILP1 groups. Knocking down CILP1 reduced the areas of scars. **D** Images of scar tissues stained by H&E staining and quantification of scar tissue areas (*n* = 6 per group). The dashed line indicated the scar tissues. Scale bar = 200 µm. **E** Images and SEI quantification of scars from AAV2-shCtrl and AAV2-shCILP1 groups (*n* = 6 per group). The long “D” and short “d” arrows stand for hypertrophic scar and normal skin tissue thicknesses, separately. “Scar elevation index” is determined by D/d ratio. Scale bar = 200 µm. **F** Collagen density exhibited by Sirius red staining. Red and yellow areas stand for COL I, while green area indicates COL III. Scale bar = 50 µm. **G**–**I** Immunofluorescence staining demonstrated that knocking down CILP1 attenuated COL I, COL III, and α-SMA proteins expression. **J** Western blot assay suggested that knocking down CILP1 decreased COL I, COL III and α-SMA protein expression (*n* = 3). Sample size is indicated as individual plots in column graphs. Results are indicated by mean ± SD. ^*^P < 0.05, ^**^P < 0.01, ^***^P < 0.001.

To investigate how CILP1 affected HS pathogenesis, a mouse HS model was constructed (Fig. 9A). Following the successful establishment of hypertrophic scarring, mice were injected with CILP1 adenovirus (AAV2-shCILP1) to knock down the expression of CILP1 (Fig. 9B). At 14 days post-modeling, scar tissues were harvested to conduct the following experiments. Gross assessment of HS size revealed that CILP1 knockdown reduced HS formation in mice at postoperative day 14 (POD 14) (Fig. 9C). Based on H&E and Masson staining, CILP1 knockdown within HS tissues decreased dermal and epidermal thicknesses, accompanied by the decreased collagen accumulation and the orderly arrangement of collagen (Fig. 9D, E). Sirius red staining displayed that CILP1 knockdown significantly reduced collagen deposition (Fig. 9F). Western blot and immunohistochemical staining assays suggested that CILP1 knockdown significantly decreased α-SMA, COL I, and COL III protein levels in the scar tissues (Fig. 9G–J).

### Recombinant human CILP1 protein promoted HS formation in vivo

**Fig. 10:**
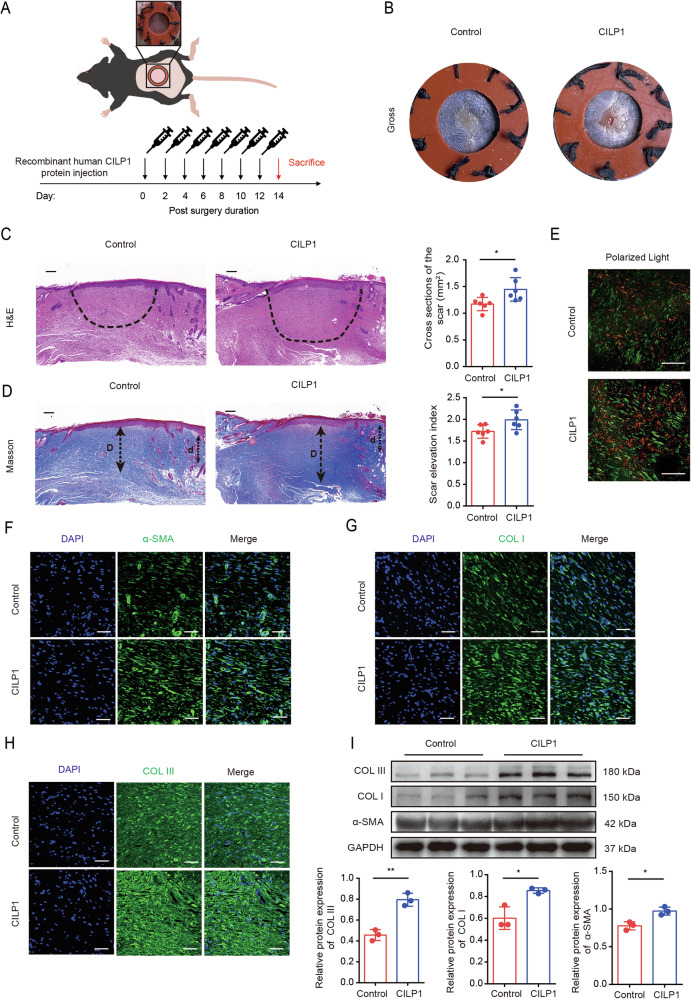
Recombinant human CILP1 protein promotes hypertrophic scar formation in C57BL/6 mice. **A** Schematic diagram of hypertrophic scar model. **B** Representative gross photographs of scars of mice from control and recombinant human CILP1 protein groups. Recombinant human CILP1 protein increased the areas of scars. **C** Images of scar tissues stained by H&E staining and quantification of scar tissue areas (*n* = 6 per group). The dashed line indicated the scar tissues. Scale bar = 200 µm. **D** Images and SEI quantification of scars in control and recombinant human CILP1 protein groups (*n* = 6 per group). The long “D” and short “d” arrows stand for hypertrophic scar and normal skin tissue thicknesses, separately. “Scar elevation index” is determined by D/d ratio. Scale bar = 200 µm. **E** Collagen density exhibited by Sirius red staining. Red and yellow areas stand for COL I, while green area indicates COL III. Scale bar = 50 µm. **F**–**H** Immunofluorescence staining demonstrated that recombinant human CILP1 protein increased COL I, COL III, and α-SMA proteins expression. **I** Western blot results showed that recombinant human CILP1 protein increased COL I, COL III, and α-SMA protein expression (*n* = 3). Sample size is indicated as individual plots in column graphs. Results are indicated by mean ± SD. ^*^P < 0.05, ^**^P < 0.01^, ***^P < 0.001.

To investigate how the recombinant human CILP1 protein affected HS development, a mouse HS model was established (Fig. 10A). The results showed that recombinant human CILP1 protein injection promoted HS formation in mice at POD 14 (Fig. 10B). Histological staining revealed that recombinant human CILP1 protein injection in wounds led to the increased thickness of dermis and epidermis, accompanied by the increase in ECM accumulation and the irregular arrangement of collagens (Fig. 10C–E). Besides, recombinant human CILP1 protein injection also elevated α-SMA expression and ECM deposition in the scar tissues of mice (Fig. 10F–I).

## Discussion

Hypertrophic scar acting as a cutaneous fibrotic disease has been studied for decades. However, there still has no ideal option for hypertrophic scar prediction, diagnosis, and treatment. According to our results, the expression of matricellular protein CILP1 increased within human HS and keloid scar tissues. Elevated expression of CILP1 was also observed in two mouse hypertrophic scar models (load-induced HS mouse model and HS mouse model) and the rabbit ear hypertrophic scar model. Corresponding to these results above, the pro-fibrotic function of CILP1 had also been confirmed in some other fibrotic diseases. CILP1 expression increased within affected human heart tissues and was related to the pathological remodeling of mouse hearts [25, 26]. Several studies previously showed that CILP1 might be the biomarker for cardiac remodeling and fibrosis associated with poor prognosis [27, 28]. Based on these studies, we assumed whether CILP1 protein was also secreted into the peripheral blood. Further, the serum CILP1 expression in HS patients increased relative to the healthy normal individuals. And the concentrations of CILP1 in the serums of patients with hypertrophic scar less than one year were higher than patients with hypertrophic scar more than one year, implying that the increase of CILP1 concentration in serums of patients indicates the occurrence of hypertrophic scarring, while the decrease of serum CILP1 level means the degradation of hypertrophic scar. Similarly, CILP1 up-regulation could be detected within blood samples in dilated cardiomyopathy patients [18]. And the serum concentrations of CILP1 in patients with cardiac fibrosis resulting from adaptive and maladaptive pulmonary hypertension were also elevated [29]. These demonstrated the increased amount of CILP1 in the diseased tissues of various human and animal skin fibrotic diseases and serums obtained from patients with hypertrophic scar, which implied the application potential of CILP1 as a candidate biomarker for human skin fibrotic diseases and a serological marker for hypertrophic scar formation and progression.

TGF-β signaling is a canonical pathway functioning in various fibrotic diseases. As one of the most important pathways in fibrosis development, it promotes hypertrophic scar formation through facilitating fibroblast to myofibroblast transition. We attempted to explore whether CILP1 interacted with TGF-β signaling to promote disease progression. According to our results, CILP1 showed major expression within human hypertrophic scar myofibroblasts, over-expressing CILP1 in HSFs or treating HSFs using exogenous recombinant human CILP1 protein can enhance HSFs proliferation, invasion, and α-SMA, COL I, and COL III levels, while knocking down CILP1 inhibited HSFs activation and attenuated α-SMA and collagens expression. Combination treatment with TGF-β1 inhibitor and si-CILP1 displayed a greater inhibitory effect on HSFs proliferation, migration, and ECM synthesis. More importantly, we found that TGF-β1 stimulation could induce HSFs CILP1 expression, and the mouse hypertrophic scar model further demonstrated the fibrosis-promotive function of CILP1 through up-regulating α-SMA and collagens expression. Consistent with our results, there were studies showing that TGF-β1 stimulation strongly elevated CILP1 expression in human cardiac fibroblasts and CILP1 promoted adverse cardiac remodeling and myocardial fibrosis by facilitating myofibroblast proliferation [18, 29]. These implied that TGF-β promotes myofibroblast activation and hypertrophic scar formation through enhancing CILP1 expression. Paradoxically, in cardiac fibrosis, CILP1 may act as a TGF-β signaling pathway antagonist, for CILP1 attenuated stress overload-induced cardiac fibrosis by interfering with TGF-β signaling [17]. We also found that knocking down CILP1 with si-CILP1 elevated the levels of TGF-β1 and p-Smad2/3 of HSFs, implying that CILP1 negatively regulated TGF-β signaling pathway. In summary, CILP1 and TGF-β pathway formed a negative feedback loop during modulating hypertrophic scar formation and progression.

Further, we explored the mechanisms of how CILP1 promotes hypertrophic scar formation. Through RNA sequencing we found that CILP1 knockdown remarkably increased the mRNA and protein expressions of PPARα, PPARδ, and PPARγ of PPARs pathway. Pull-down assay plus mass spectrometry (Pull-down-MS), molecular docking, GST pull-down and Co-IP assay confirmed the direct protein interaction between CILP1 and YBX1. CILP1 regulates gene expression by binding to transcription factors YBX1. YBX1 belongs to the YBX family, previous study has revealed its association with multiple fibrosis-related processes through transcriptionally regulating gene expression [22–24]. YBX1 promotes the expression of α-SMA and p-Smad2 along with the progression of human buccal mucosal fibroblasts to myofibroblasts transition in oral submucous fibrosis [30], which correlates with TGF-β signaling pathway. In lung adenocarcinoma, TGF-β1 can act as the upstream regulator of YBX1 to participate in the modulation of epithelial-to-mesenchymal transition (EMT), TGF-β1 treatment stimulates YBX1 expression and facilitates the nuclear translocation of cytosolic YBX1, which then upregulates the expression of mesenchymal markers in A549 cells [31]. Besides, TGF-β1 treatment induces YBX1 nuclear translocation in hepatic progenitor cells (HPCs) and promotes the proliferation of HPCs in liver fibrosis [32]. A subsequent study showed that YBX1 promotes HPCs proliferation, EMT, and ECM deposition during liver fibrosis [33]. In nasopharyngeal carcinoma (NPC), the expression of YBX1 positively correlates with Vimentin expression, and TGF-β1 stimulation promotes YBX1 expression and EMT within CNE1 cells [34]. Besides, recent studies demonstrated that YBX1 can be shuttled between the cytoplasm and the nucleus, and various interacting proteins such as GSDME and rSjp40 can induce the nuclear translocation of YBX1 [35, 36]. In our study, we proved that CILP1 directly bound to YBX1 and markedly promoted YBX1 nuclear translocation of HSFs. In our study, we found that TGF-β1 induced CILP1 expression and CILP1 promoted YBX1 nuclear translocation by directly bound to YBX1. These implied that during hypertrophic scar formation, CILP1 may have mediated TGF-β1-stimulation-induced YBX1 nuclear translocation by forming a protein complex with YBX1, which may benefit filling in the gap of how TGF-β1 stimulates YBX1 nuclear translocation in various diseases. We further confirmed that YBX1 knockdown remarkably elevates the expression of PPARα, PPARδ, and PPARγ at both the mRNA and protein levels. ChIP-qPCR and luciferase reporter assay results validated the binding of YBX1 to the promoters of PPARα, PPARδ, and PPARγ. In conclusion, we firstly demonstrated that CILP1 harbors the ability to induce YBX1 nuclear translocation, and CILP1 can inhibit PPARs expression through directly interacting with YBX1 in HSFs, thus aggravating HS formation.

PPARs are ligand-activated transcription factors belonging to the nuclear hormone receptor family, which can modulate various physiological processes [37–39]. Three different isoforms of PPARs have been identified: PPARα, PPARδ, and PPARγ [40]. In our study, we found that the expression of PPARs was down-regulated in human hypertrophic scar tissues. Inhibiting PPARs including PPARα, PPARδ, and PPARγ with corresponding inhibitor facilitated HSFs proliferation and migration and up-regulated the expression of α-SMA, COL I, and COL III in HSFs, and PPARs pan-inhibitor Norathyriol exhibits a better effect on promoting HSFs ECM synthesis than individual inhibitors. Evidence is accumulating that the PPARs are crucial for modulating skin fibrosis-associated processes [41, 42]. The up-regulation of PPARα has been shown to be involved in repressing the collagen I-promoted 3T3-L1 preadipocyte migration [43]. And the interstitial tissues of aged PPARα−/− mice kidneys exhibiting increased COL I expression and impaired renal PPARα pathway [44]. Besides, existing study revealed that knocking down PPARβ/δ in fibroblasts facilitated fibroblast to myofibroblast transition and resulted in an elevated activation of TGFβRII and SMAD3. PPARβ/δ activation by GW501516 attenuated the α-SMA expression in SSc fibroblasts [45]. Regarding to hypertrophic scar, PPAR-γ agonist treatment decreased the expression of ECM related genes including COL I, Fibronectin (FN), Connective tissue growth factor (CTGF), TGF-β1, and Smad3 in HSFBs [46–48]. In the lesional skin tissues and fibroblasts from human systemic sclerosis (SSc) patients, the expression of PPARγ was decreased, further study confirmed that TGF-β treatment contributed to its diminished expression [49]. In the liver, PPARγ reduction induced activation of quiescent hepatic stellate cells and restoration of PPARγ reversed this process with inhibition of the known activation markers such as α-SMA, collagen, and TGF-β expression [50]. These are in agreement with our study and confirm that PPARs activation may contribute to the improvement of fibrosis diseases including hypertrophic scar. In contrast, there were also studies showing that PPARγ can disrupt the downstream fibrotic response induced by the intracellular TGF-β/Smad signaling in skin fibroblasts [51]. PPARγ deletion led to an increased risk of bleomycin-mediated skin fibrosis, and PPARγ-specific agonists can mitigate the cutaneous sclerosis severity [52, 53]. Furthermore, pan-PPAR agonists have been widely shown to improve in vivo fibrosis models of skin fibrosis [54]. These suggested that CILP1 promotes hypertrophic scar formation through YBX1 mediating PPARs transcription suppression.

Except the finding on CILP1, CILP2 is also involved in hypertrophic scar formation [55]. Both CILP1 and CILP2 belong to the cartilage intermediate layer proteins, they share a lot of similarities in protein structure and can be cleaved to the C- and N-terminal fragments at the furin endoprotease consensus site [17]. Clinically, they harbor the potential to serve as serological biomarkers for HS development [55]. Biologically, each of them can exacerbate the profibrotic phenotype by promoting HSFs cell proliferation, migration, myofibroblast activation, and collagen synthesis. Nevertheless, they exhibit different mechanisms of action in the formation of hypertrophic scar. CILP1 exerted its function by forming a negative feedback loop with TGF-β and inhibited the transcription of PPARs via interaction with YBX1, while CILP2 acts through interacting with ACLY and attenuating ACLY ubiquitination, thus prompting Snail acetylation [55]. These demonstrated that CILP1 and CILP2 share similarities in protein structure and biological behaviors, but they promote hypertrophic scar formation through distinct mechanisms.

**Fig. 11:**
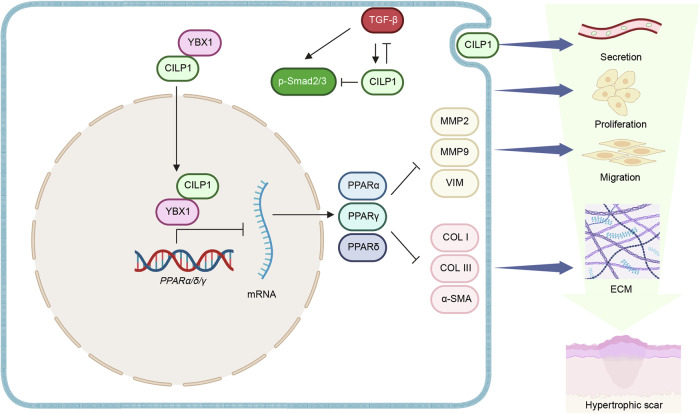
Schematic diagram of the proposed mechanism. CILP1 was upregulated in hypertrophic scar and harbored potential to be a biomarker for hypertrophic scar. CILP1 was involved in a negative feedback loop with TGF-β and inhibited the transcription of PPARs via interaction with YBX1. This interaction promoted cell proliferation, migration, and collagen production in HSFs.

In summary, our study highlights the critical role of CILP1, especially the intracellular CILP1 in the pathogenesis of HS and potentially other fibrotic diseases. We have demonstrated that CILP1 is significantly upregulated in both human and animal models of skin fibrosis, and its increased serum levels in HS patients suggest its utility as a novel biomarker for early diagnosis and progression monitoring. Furthermore, the interaction of CILP1 with TGF-β and YBX1 unveils a complex regulatory network that not only enhances our understanding of fibrotic mechanisms but also opens new avenues for targeted therapeutic strategies. By modulating this pathway, particularly through the suppression of PPARs signaling, CILP1 facilitates fibroblast activation and exacerbates fibrotic responses (Fig. 11). However, the absence of the precise mechanism of how extracellular CILP1 modulates hypertrophic scar formation may serve as a limitation to the present study. To sum up, targeting CILP1 offers a promising approach to mitigate hypertrophic scar formation and treat other related fibrotic conditions. Our findings provide a foundation for future research aimed at developing effective interventions especially CILP1 specific inhibitors for skin fibrosis, potentially transforming patient outcomes in fibrotic diseases.

## Supplementary information

Supplementary information

Full and uncropped western blots

## Data Availability

The datasets used and/or analyzed during the current study are available from the corresponding author on reasonable request.

## References

[1] Moortgat P, Meirte J, Maertens K, Lafaire C, De Cuyper L, Anthonissen M. Can a Cohesive Silicone Bandage Outperform an Adhesive Silicone Gel Sheet in the Treatment of Scars? A Randomized Comparative Trial. Plast Reconstr Surg. 2019;143:902–11. 30601234 10.1097/PRS.0000000000005369

[2] Frangogiannis N. Transforming growth factor-beta in tissue fibrosis. J Exp Med. 2020;217:e20190103. 10.1084/jem.20190103 PMC7062524 32997468

[3] Gao Y, Liu Y, Zheng D, Ho C, Wen D, Sun J, et al. HDAC5-mediated Smad7 silencing through MEF2A is critical for fibroblast activation and hypertrophic scar formation. Int J Biol Sci. 2022;18:5724–39. 36263180 10.7150/ijbs.76140 PMC9576526

[4] Sen CK, Gordillo GM, Roy S, Kirsner R, Lambert L, Hunt TK, et al. Human skin wounds: a major and snowballing threat to public health and the economy. Wound Repair Regen. 2009;17:763–71. 19903300 10.1111/j.1524-475X.2009.00543.x PMC2810192

[5] Gold MH, Berman B, Clementoni MT, Gauglitz GG, Nahai F, Murcia C. Updated international clinical recommendations on scar management: part 1-evaluating the evidence. Dermatol Surg. 2014;40:817–24. 25068543 10.1111/dsu.0000000000000049

[6] Gauglitz GG. Management of keloids and hypertrophic scars: current and emerging options. Clin Cosmet Inv Derm. 2013;6:103–14. 10.2147/CCID.S35252 PMC3639020 23637546

[7] Wang J, Zhao M, Zhang H, Yang F, Luo L, Shen K, et al. KLF4 Alleviates Hypertrophic Scar Fibrosis by Directly Activating BMP4 Transcription. Int J Biol Sci. 2022;18:3324–36. 35637963 10.7150/ijbs.71167 PMC9134901

[8] Gras C, Ratuszny D, Hadamitzky C, Zhang H, Blasczyk R, Figueiredo C. miR-145 Contributes to Hypertrophic Scarring of the Skin by Inducing Myofibroblast Activity. Mol Med. 2015;21:296–304. 25876136 10.2119/molmed.2014.00172 PMC4503650

[9] Douglass A, Wallace K, Parr R, Park J, Durward E, Broadbent I, et al. Antibody-targeted myofibroblast apoptosis reduces fibrosis during sustained liver injury. J Hepatol. 2008;49:88–98. 18394744 10.1016/j.jhep.2008.01.032

[10] Liang X, Chai B, Duan R, Zhou Y, Huang X, Li Q. Inhibition of FKBP10 Attenuates Hypertrophic Scarring through Suppressing Fibroblast Activity and Extracellular Matrix Deposition. J Invest Dermatol. 2017;137:2326–35. 28774593 10.1016/j.jid.2017.06.029

[11] Yin SL, Qin ZL, Yang X. Role of periostin in skin wound healing and pathologic scar formation. Chinese Med J Peking. 2020;133:2236–8. 10.1097/CM9.0000000000000949 PMC7508428 32769490

[12] Nikoloudaki G, Creber K, Hamilton DW. Wound healing and fibrosis: a contrasting role for periostin in skin and the oral mucosa. Am J Physiol-Cell Ph. 2020;318:C1065–77. 10.1152/ajpcell.00035.2020 PMC7311745 32267719

[13] Crawford J, Nygard K, Gan BS, O’Gorman DB. Periostin induces fibroblast proliferation and myofibroblast persistence in hypertrophic scarring. Exp Dermatol. 2015;24:120–6. 25421393 10.1111/exd.12601

[14] He J, Feng C, Sun J, Lu K, Chu T, Zhou Y, et al. Cartilage intermediate layer protein is regulated by mechanical stress and affects extracellular matrix synthesis. Mol Med Rep. 2018;17:6130–7. 29436660 10.3892/mmr.2018.8588

[15] Wu T, Zhang Q, Wu S, Hu W, Zhou T, Li K, et al. CILP-2 is a novel secreted protein and associated with insulin resistance. J Mol Cell Biol. 2019;11:1083–94. 30896018 10.1093/jmcb/mjz016 PMC6934158

[16] van Nieuwenhoven FA, Munts C, Op’T VR, Gonzalez A, Diez J, Heymans S, et al. Cartilage intermediate layer protein 1 (CILP1): A novel mediator of cardiac extracellular matrix remodelling. Sci Rep. 2017;7:16042. 10.1038/s41598-017-16201-y PMC5700204 29167509

[17] Zhang CL, Zhao Q, Liang H, Qiao X, Wang JY, Wu D, et al. Cartilage intermediate layer protein-1 alleviates pressure overload-induced cardiac fibrosis via interfering TGF-beta1 signaling. J Mol Cell Cardiol. 2018;116:135–44. 29438665 10.1016/j.yjmcc.2018.02.006

[18] Zhang QJ, He Y, Li Y, Shen H, Lin L, Zhu M, et al. Matricellular Protein Cilp1 Promotes Myocardial Fibrosis in Response to Myocardial Infarction. Circ Res. 2021;129:1021–35. 34610755 10.1161/CIRCRESAHA.121.319482 PMC8722455

[19] Aarabi S, Bhatt KA, Shi Y, Paterno J, Chang EI, Loh SA, et al. Mechanical load initiates hypertrophic scar formation through decreased cellular apoptosis. FASEB J. 2007;21:3250–61. 17504973 10.1096/fj.07-8218com

[20] Griffin MF, Borrelli MR, Garcia JT, Januszyk M, King M, Lerbs T, et al. JUN promotes hypertrophic skin scarring via CD36 in preclinical in vitro and in vivo models. Sci Transl Med. 2021;13:eabb3312. 10.1126/scitranslmed.abb3312 PMC8988368 34516825

[21] Song B, Zhang W, Guo S, Han Y, Zhang Y, Ma F, et al. Adenovirus-mediated METH1 gene expression inhibits hypertrophic scarring in a rabbit ear model. Wound Repair Regen. 2009;17:559–68. 19614921 10.1111/j.1524-475X.2009.00514.x

[22] Lyabin DN, Eliseeva IA, Ovchinnikov LP. YB-1 protein: functions and regulation. Wires Rna. 2014;5:95–110. 24217978 10.1002/wrna.1200

[23] Tang Z, Lin B, Li W, Li X, Liu F, Zhu X. Y-box binding protein 1 promotes chromatin accessibility to aggravate liver fibrosis. Cell Signal. 2023;109:110750. 37290675 10.1016/j.cellsig.2023.110750

[24] Bernhardt A, Fehr A, Brandt S, Jerchel S, Ballhause TM, Philipsen L, et al. Inflammatory cell infiltration and resolution of kidney inflammation is orchestrated by the cold-shock protein Y-box binding protein-1. Kidney Int. 2017;92:1157–77. 28610763 10.1016/j.kint.2017.03.035

[25] Park S, Ranjbarvaziri S, Zhao P, Ardehali R. Cardiac Fibrosis Is Associated With Decreased Circulating Levels of Full-Length CILP in Heart Failure. JACC Basic Transl Sci. 2020;5:432–43. 32478206 10.1016/j.jacbts.2020.01.016 PMC7251193

[26] Kalyanasundaram A, Li N, Gardner ML, Artiga EJ, Hansen BJ, Webb A, et al. Fibroblast-Specific Proteotranscriptomes Reveal Distinct Fibrotic Signatures of Human Sinoatrial Node in Nonfailing and Failing Hearts. Circulation. 2021;144:126–43. 33874740 10.1161/CIRCULATIONAHA.120.051583 PMC8277727

[27] Keranov S, Jafari L, Haen S, Vietheer J, Kriechbaum S, Dorr O, et al. CILP1 as a biomarker for right ventricular dysfunction in patients with ischemic cardiomyopathy. Pulm Circ. 2022;12:e12062. 10.1002/pul2.12062 PMC9052998 35506075

[28] Wang C, Jian W, Luo Q, Cui J, Qing Y, Qin C, et al. Prognostic value of cartilage intermediate layer protein 1 in chronic heart failure. Esc Heart Fail. 2022;9:345–52. 34939356 10.1002/ehf2.13746 PMC8787959

[29] Keranov S, Dorr O, Jafari L, Troidl C, Liebetrau C, Kriechbaum S, et al. CILP1 as a biomarker for right ventricular maladaptation in pulmonary hypertension. Eur Respir J. 2021;57:1901192. 33184116 10.1183/13993003.01192-2019

[30] Yu CH, Fang CY, Yu CC, Hsieh PL, Liao YW, Tsai LL, et al. LINC00312/YBX1 Axis Regulates Myofibroblast Activities in Oral Submucous Fibrosis. Int J Mol Sci. 2020;21:2979. 32340273 10.3390/ijms21082979 PMC7215884

[31] Ha B, Lee EB, Cui J, Kim Y, Jang HH. YB-1 overexpression promotes a TGF-beta1-induced epithelial-mesenchymal transition via Akt activation. Biochem Bioph Res Co. 2015;458:347–51. 10.1016/j.bbrc.2015.01.114 25645014

[32] Guo Y, Zhu J, Xu X, Shen B, Shen Z, Li B, et al. TGF-beta/YB-1/Atg7 axis promotes the proliferation of hepatic progenitor cells and liver fibrogenesis. BBA Mol Basis Dis. 2022;1868:166290. 10.1016/j.bbadis.2021.166290 34662704

[33] Guo Y, Xu X, Dong H, Shen B, Zhu J, Shen Z, et al. Loss of YB-1 alleviates liver fibrosis by suppressing epithelial-mesenchymal transition in hepatic progenitor cells. BBA Mol Basis Dis. 2022;1868:166510. 10.1016/j.bbadis.2022.166510 35926755

[34] Zhou LL, Ni J, Feng WT, Yao R, Yue S, Zhu YN, et al. High YBX1 expression indicates poor prognosis and promotes cell migration and invasion in nasopharyngeal carcinoma. Exp Cell Res. 2017;361:126–34. 29024700 10.1016/j.yexcr.2017.10.009

[35] Lv J, Liu Y, Mo S, Zhou Y, Chen F, Cheng F, et al. Gasdermin E mediates resistance of pancreatic adenocarcinoma to enzymatic digestion through a YBX1-mucin pathway. Nat Cell Biol. 2022;24:364–72. 35292781 10.1038/s41556-022-00857-4 PMC8924000

[36] Chen L, Zhou Q, Liu E, Zhang J, Duan L, Zhu D, et al. rSjp40 inhibits activated hepatic stellate cells by promoting nuclear translocation of YB1 and inducing BMP-7/Smad1/5/8 pathway. Parasite Vector. 2019;12:279. 10.1186/s13071-019-3539-z PMC6545069 31151477

[37] Chinetti G, Fruchart JC, Staels B. Peroxisome proliferator-activated receptors (PPARs): nuclear receptors at the crossroads between lipid metabolism and inflammation. Inflamm Res. 2000;49:497–505. 11089900 10.1007/s000110050622

[38] Wahli W, Michalik L. PPARs at the crossroads of lipid signaling and inflammation. Trends Endocrin Met. 2012;23:351–63. 10.1016/j.tem.2012.05.001 22704720

[39] Christofides A, Konstantinidou E, Jani C, Boussiotis VA. The role of peroxisome proliferator-activated receptors (PPAR) in immune responses. Metabolism. 2021;114:154338. 10.1016/j.metabol.2020.154338 PMC7736084 32791172

[40] Lakatos HF, Thatcher TH, Kottmann RM, Garcia TM, Phipps RP, Sime PJ. The Role of PPARs in Lung Fibrosis. Ppar Res. 2007;2007:71323. 17710235 10.1155/2007/71323 PMC1940051

[41] Dantas AT, Pereira MC, de Melo RM, Da RLJ, Pitta IR, Marques CD, et al. The Role of PPAR Gamma in Systemic Sclerosis. PPAR Res. 2015;2015:124624. 26064084 10.1155/2015/124624 PMC4438188

[42] Varkey M, Ding J, Tredget EE. Differential collagen-glycosaminoglycan matrix remodeling by superficial and deep dermal fibroblasts: potential therapeutic targets for hypertrophic scar. Biomaterials. 2011;32:7581–91. 21802722 10.1016/j.biomaterials.2011.06.070

[43] Liu X, Xu Q, Long X, Liu W, Zhao Y, Hayashi T, et al. Silibinin-induced autophagy mediated by PPARalpha-sirt1-AMPK pathway participated in the regulation of type I collagen-enhanced migration in murine 3T3-L1 preadipocytes. Mol Cell Biochem. 2019;450:1–23. 29916120 10.1007/s11010-018-3368-y

[44] Chung KW, Lee EK, Lee MK, Oh GT, Yu BP, Chung HY. Impairment of PPARalpha and the Fatty Acid Oxidation Pathway Aggravates Renal Fibrosis during Aging. J Am Soc Nephrol. 2018;29:1223–37. 29440279 10.1681/ASN.2017070802 PMC5875952

[45] Sng MK, Chan J, Teo Z, Phua T, Tan E, Wee J, et al. Selective deletion of PPARbeta/delta in fibroblasts causes dermal fibrosis by attenuated LRG1 expression. Cell Discov. 2018;4:15. 29619245 10.1038/s41421-018-0014-5 PMC5880809

[46] Zhu HY, Li C, Zheng Z, Zhou Q, Guan H, Su LL, et al. Peroxisome proliferator-activated receptor-gamma (PPAR-gamma) agonist inhibits collagen synthesis in human hypertrophic scar fibroblasts by targeting Smad3 via miR-145. Biochem Bioph Res Co. 2015;459:49–53. 10.1016/j.bbrc.2015.02.061 25704091

[47] Zhang GY, Cheng T, Zheng MH, Yi CG, Pan H, Li ZJ, et al. Peroxisome proliferator-activated receptor-gamma (PPAR-gamma) agonist inhibits transforming growth factor-beta1 and matrix production in human dermal fibroblasts. J Plast Reconstr Aes. 2010;63:1209–16. 10.1016/j.bjps.2009.06.032 19617014

[48] Zhang GY, Cheng T, Zheng MH, Yi CG, Pan H, Li ZJ, et al. Activation of peroxisome proliferator-activated receptor-gamma inhibits transforming growth factor-beta1 induction of connective tissue growth factor and extracellular matrix in hypertrophic scar fibroblasts in vitro. Arch Dermatol Res. 2009;301:515–22. 19466435 10.1007/s00403-009-0959-1

[49] Wei J, Ghosh AK, Sargent JL, Komura K, Wu M, Huang QQ, et al. PPARgamma downregulation by TGFss in fibroblast and impaired expression and function in systemic sclerosis: a novel mechanism for progressive fibrogenesis. Plos One. 2010;5:e13778. 21072170 10.1371/journal.pone.0013778 PMC2970611

[50] Hazra S, Xiong S, Wang J, Rippe RA, Krishna V, Chatterjee K, et al. Peroxisome proliferator-activated receptor gamma induces a phenotypic switch from activated to quiescent hepatic stellate cells. J Biol Chem. 2004;279:11392–401. 14702344 10.1074/jbc.M310284200

[51] Ghosh AK, Bhattacharyya S, Lakos G, Chen SJ, Mori Y, Varga J. Disruption of transforming growth factor beta signaling and profibrotic responses in normal skin fibroblasts by peroxisome proliferator-activated receptor gamma. Arthritis Rheum. 2004;50:1305–18. 15077315 10.1002/art.20104

[52] Sha W, Thompson K, South J, Baron M, Leask A. Loss of PPARgamma expression by fibroblasts enhances dermal wound closure. Fibrogenesis Tissue Repair. 2012;5:5. 22502865 10.1186/1755-1536-5-5 PMC3348009

[53] Kapoor M, McCann M, Liu S, Huh K, Denton CP, Abraham DJ, et al. Loss of peroxisome proliferator-activated receptor gamma in mouse fibroblasts results in increased susceptibility to bleomycin-induced skin fibrosis. Arthritis Rheum. 2009;60:2822–9. 19714649 10.1002/art.24761

[54] Ruzehaji N, Frantz C, Ponsoye M, Avouac J, Pezet S, Guilbert T, et al. Pan PPAR agonist IVA337 is effective in prevention and treatment of experimental skin fibrosis. Ann Rheum Dis. 2016;75:2175–83. 26961294 10.1136/annrheumdis-2015-208029 PMC5136696

[55] Wang J, Du J, Wang Y, Song Y, Wu J, Wang T, et al. CILP2 promotes hypertrophic scar through Snail acetylation by interaction with ACLY. BBA Mol Basis Dis. 2024;1870:167202. 10.1016/j.bbadis.2024.167202 38670440

