# Supplementary material for: CILP1 interacting with YBX1 promotes hypertrophic scar formation by suppressing PPARs transcription
Source: Cell Death Dis. 2025 May 9;16(1):371. doi: 10.1038/s41419-025-07554-8 (PMC12064789; doi:10.1038/s41419-025-07554-8)
Supplement: Supplementary file 1 — Supplementary information [file 41419_2025_7554_MOESM1_ESM.pdf]

CILP1 interacting with YBX1 promotes hypertrophic scar formation by suppressing PPARs transcription

Jianzhang Wang[1#], Juan Du[2#], Yajuan Song[1#], Xiaoying Tan[3], Junzheng Wu[1], Tong Wang[1], Yi Shi[1], Xingbo Xu[4*], Zhou Yu[1*], and Baoqiang Song[1*]

1. Department of Plastic Surgery, Xijing Hospital, Fourth Military Medical University (Air Force Medical University), Xi'an 710032, China.

2. Department of Dermatology, Xuanwu Hospital, Capital Medical University, Beijing, 100053, China.

3. Department of Nephrology and Rheumatology, University Medical Center Göttingen, Robert-Koch-Str. 40, 37075 Göttingen, Germany.

4. Clinic for Cardiology and Pulmonology, University Medical Center Göttingen, Robert-Koch-Str. 40, 37075 Göttingen, Germany.

Supplementary Materials

Supplementary Figures

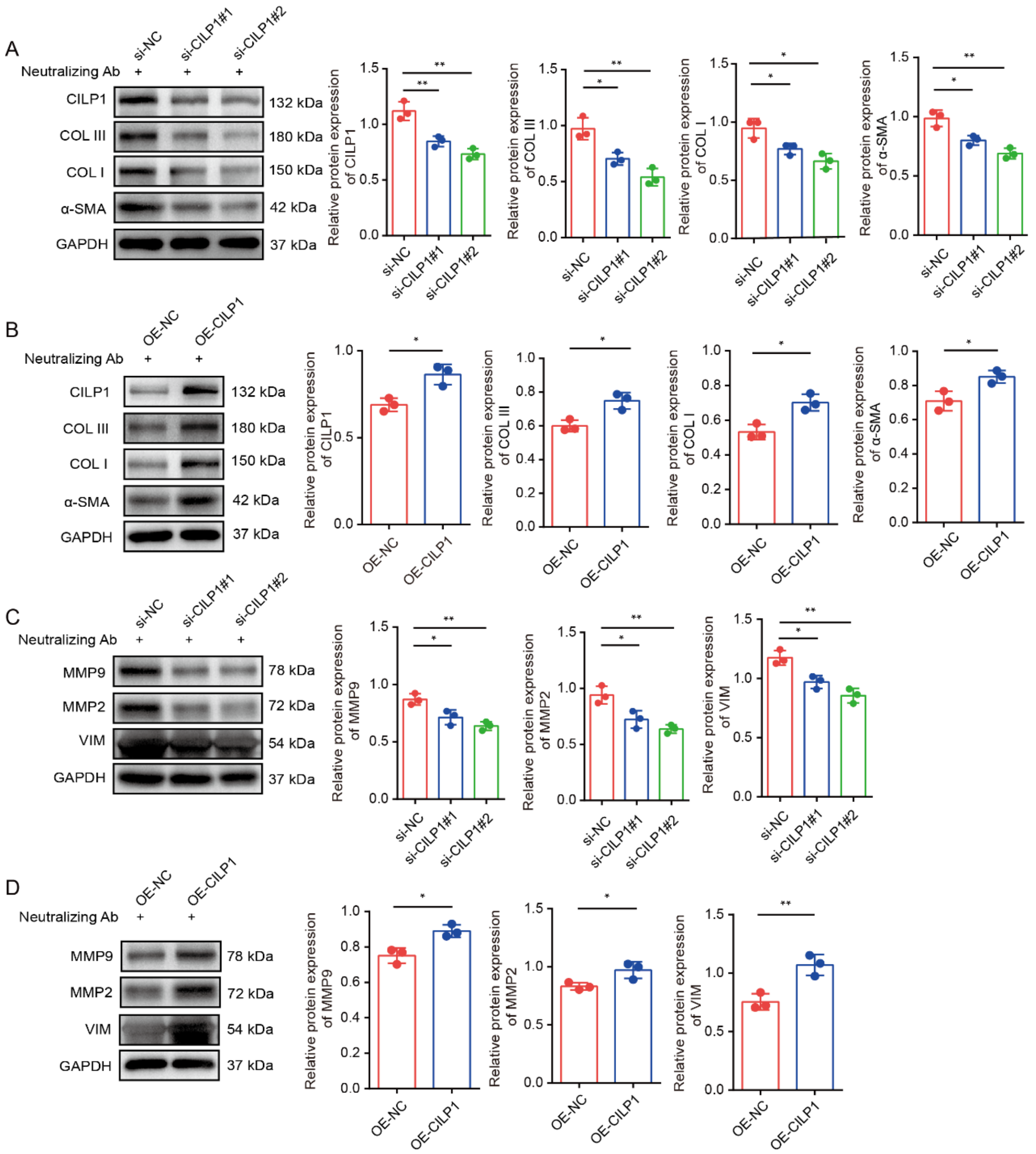

Supplementary Fig. 1 Intracellular CILP1 promoted HSFs activation, migration, and extracellular matrix synthesis using antibody neutralization test. A Western blot assay tested the protein expressions of COL I, COL III, and α-SMA in HSFs transfected with si-NC or CILP1 siRNAs incubated with CILP1 neutralizing antibody in conditioned medium (n = 3). **B** Results of Western blot showing the protein levels of

COL I, COL III, and α-SMA in HSFs transfected with OE-NC or OE-CILP1 incubated with CILP1 neutralizing antibody in conditioned medium (n = 3). **C** Western blot assay tested the protein expressions of MMP9, MMP2, and VIM in HSFs transfected with si-NC or CILP1 siRNAs incubated with CILP1 neutralizing antibody in conditioned medium. **D** Results of Western blot exhibiting the protein expressions of MMP9, MMP2, and VIM in HSFs transfected with OE-NC or OE-CILP1 incubated with CILP1 neutralizing antibody in conditioned medium (n = 3). Sample size is indicated as individual plots in column graphs. Results are indicated by mean ± SD. $^{*}P < 0.05$, $^{**}P < 0.01$.

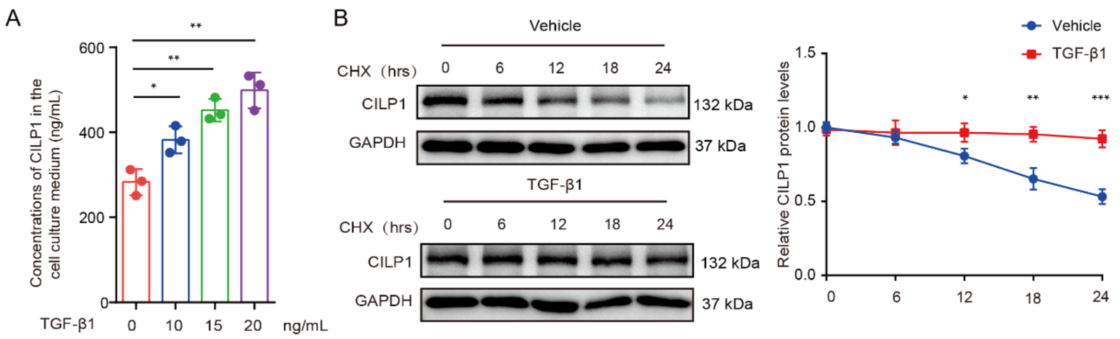

Supplementary Fig. 2 TGF-β1 stimulation elevated CILP1 protein levels by enhancing CILP1 protein stability. A ELISA assays showed the extracellular CILP1 levels in the culture medium of HSFs after TGF-β1 (0, 10, 15, 20 ng/mL) stimulation for 48 h (n = 3). **B** Western blot assay detected the protein levels of CILP1 in HSFs treated with TGF-β1 (0, 10 ng/mL) combined with CHX (20 μg/mL) for the indicated times (n = 3). Sample size is indicated as individual plots in column graphs. Results are indicated by mean ± SD. $^{*}P < 0.05$, $^{**}P < 0.01$, $^{***}P < 0.001$.

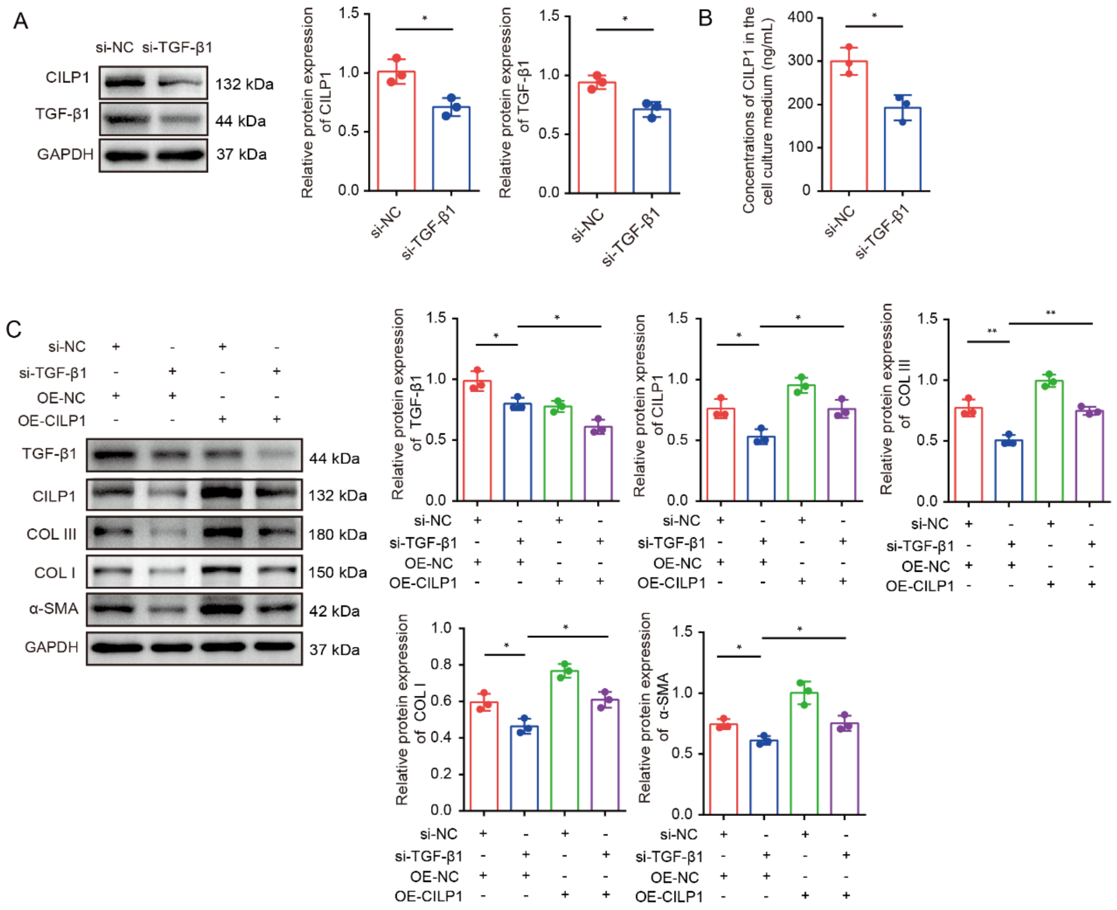

Supplementary Fig. 3 CILP1 and TGF-β pathway formed a negative feedback loop in HSFs. A Western blot results showed that knocking down TGF-β1 with si-TGF-β1 attenuated CILP1 expression in HSFs (n = 3). **B** The results of ELISA assay showed that knocking down TGF-β1 with si-TGF-β1 decreased the amount of extracellular CILP1 in the culture medium of HSFs (n = 3). **C** The protein expressions of TGF-β1, CILP1, α-SMA, COL I, and COL III in HSFs suffered from si-TGF-β1 and/ or CILP1 overexpression plasmid transfection (n = 3). Sample size is indicated as individual plots in column graphs. Results are indicated by mean ± SD. $^{*}P < 0.05$, $^{**}P < 0.01$.

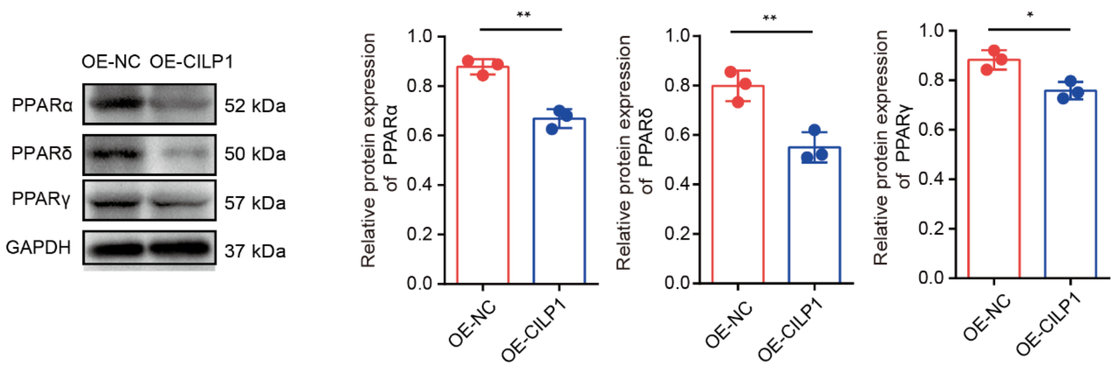

Supplementary Fig. 4 CILP1 suppressed PPARs expression in HSFs. The results of Western blot showed that CILP1 overexpression decreased the protein expression of PPARα, PPARδ, and PPARγ in HSFs (n = 3). Sample size is indicated as individual plots in column graphs. Results are indicated by mean ± SD. $^{*}P < 0.05$, $^{**}P < 0.01$.

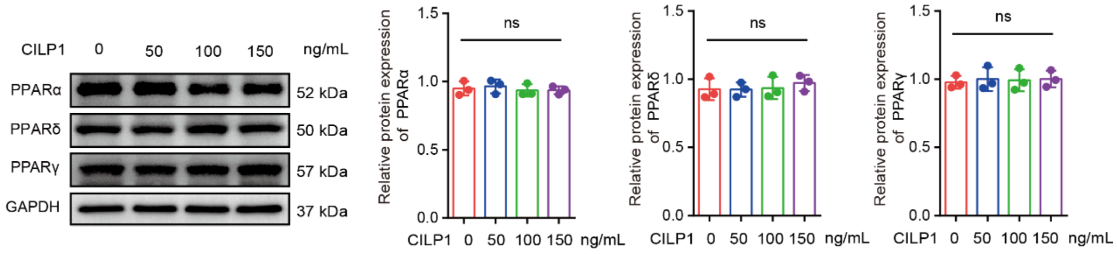

Supplementary Fig. 5 Recombinant human CILP1 protein treatment failed to stimulate the protein expression of PPARα, PPARδ, and PPARγ in HSFs. Western blot assay detected PPARα, PPARδ, and PPARγ levels in HSFs treated with recombinant human CILP1 protein (0, 50, 100, 150 ng/mL) (n = 3). Sample size is indicated as individual plots in column graphs. Results are indicated by mean ± SD. ns indicates not significant.

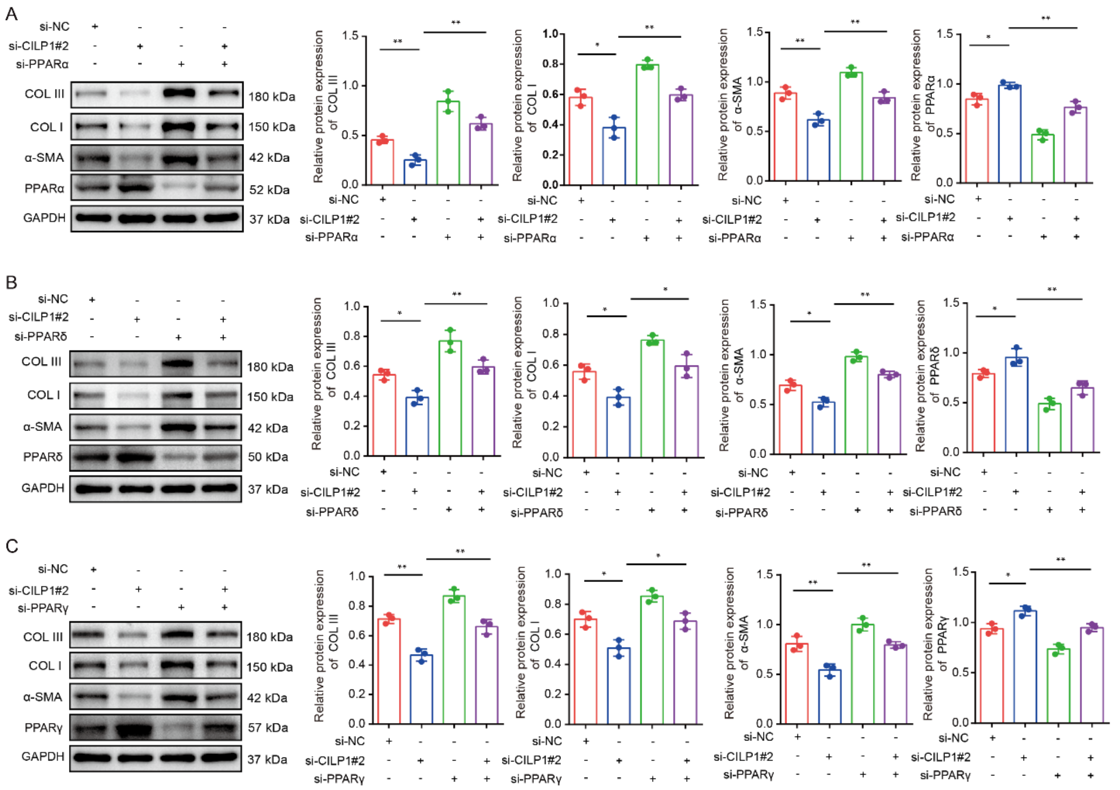

Supplementary Fig. 6 Inhibiting PPARs pathway enhanced the profibrotic effects of CILP1 in HSFs. A The protein expressions of PPARα, COL I, COL III, and α-SMA in HSFs after being treated with si-CILP1#2 or/and si- PPARα detected by Western blot (n = 3). **B** The protein expressions of PPARδ, COL I, COL III, and α-SMA protein expression in HSFs after being treated with si-CILP1#2 or/and si-PPARδ detected by Western blot (n = 3). **C** The protein expressions of PPARγ, COL I, COL III, and α-SMA in HSFs after being treated with si-CILP1#2 or/and si- PPARγ detected by Western blot (n = 3). Sample size is indicated as individual plots in column graphs. Results are indicated by mean ± SD. $^{*}P < 0.05$, $^{**}P < 0.01$.

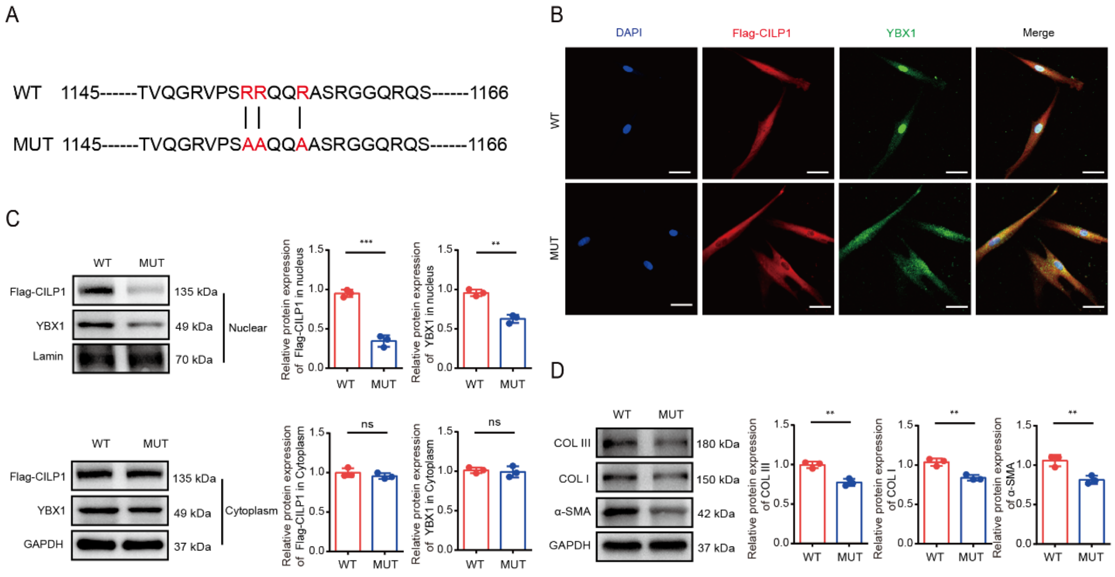

Supplementary Fig. 7 CILP1 modulates YBX1 nuclear import. A The nuclear localization sequence (NLS) of CILP1 and the mutated amino acids in NLS of CILP1 protein. **B, C** Immunofluorescence staining and Western blot assay demonstrated that mutations in the NLS sequence of CILP1 could attenuate the nuclear location of CILP1 and YBX1 in HSFs. Scale bar = 50 μm. **D** The results of Western blot showed the expression of α-SMA, COL I, and COL III in HSFs after the WT and MUT CILP1 plasmid transfection. Sample size is indicated as individual plots in column graphs. Results are indicated by mean ± SD. ns indicates not significant, $^{**}P < 0.01$. $^{***}P < 0.001$.

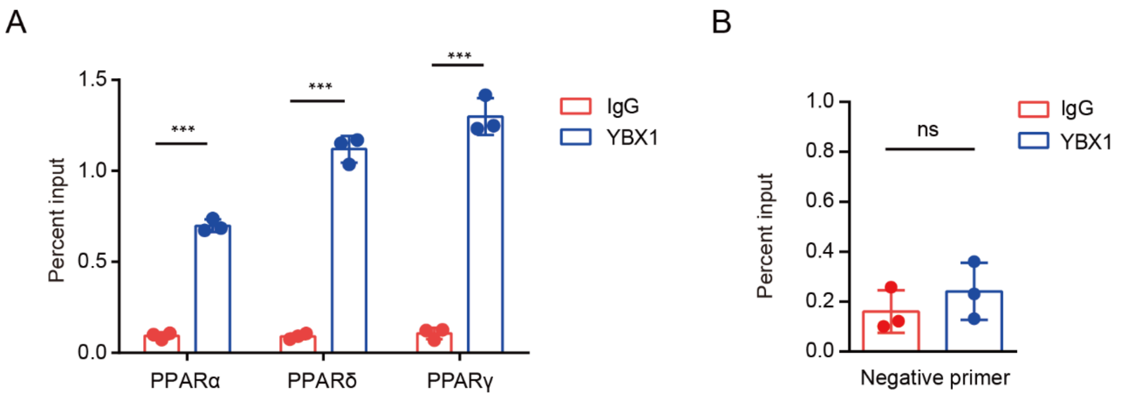

Supplementary Fig. 8 ChIP-qPCR confirmed the binding of YBX1 to the promoters of PPARα, PPARδ, and PPARγ. A ChIP-qPCR confirmed the binding of YBX1 to the promoter regions of PPARα, PPARδ, and PPARγ. **B** The results of ChIP-qPCR amplification using negative primers which did not match the promoter regions of PPARα, PPARδ, and PPARγ excluded the non-specific binding of YBX1 protein to the DNA sequences of other genes rather than PPARα, PPARδ, and PPARγ. Sample size is indicated as individual plots in column graphs. Results are indicated by mean ± SD. ns indicates not significant, $^{***}P < 0.001$.

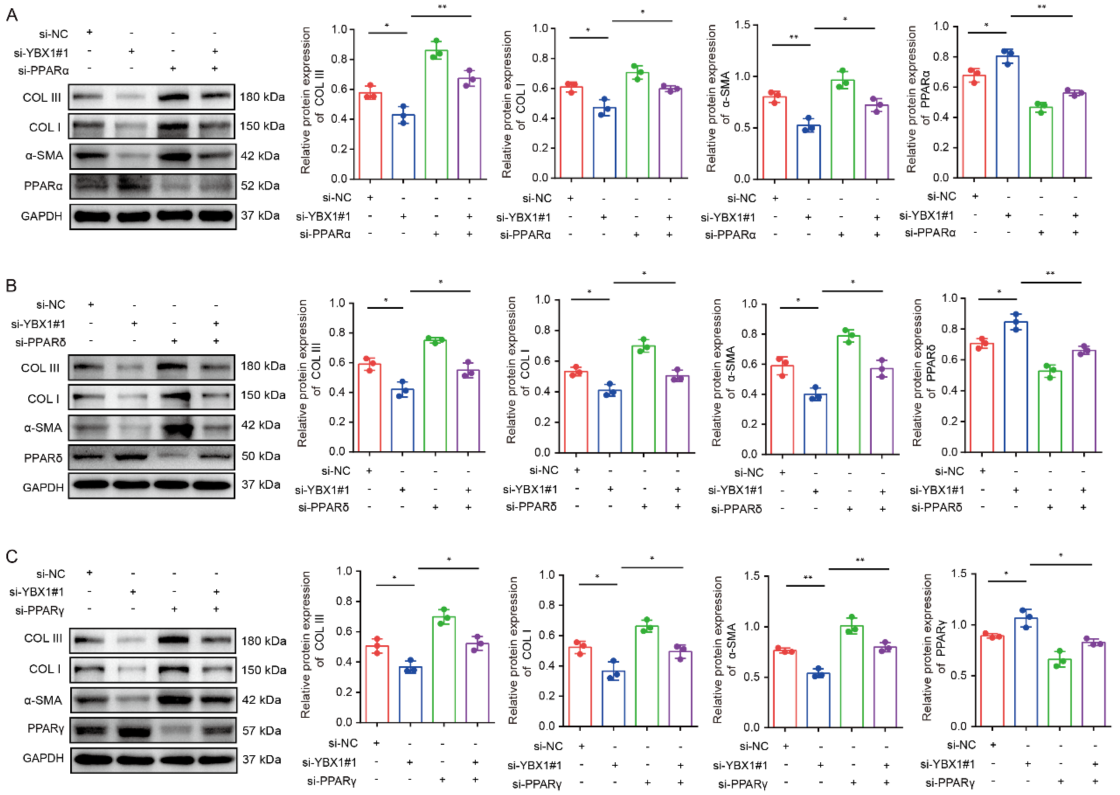

Supplementary Fig. 9 PPARs knockdown reversed YBX1 knockdown-induced downregulation of COL I, COL III, and α-SMA in HSFs. A PPARα, COL I, COL III, and α-SMA protein expression within HSFs after being treated with si-YBX1#1 or/and si- PPARα detected by Western blot (n = 3). **B** PPARδ, COL I, COL III, and α-SMA protein expression within HSFs after being treated with si-YBX1#1 or/and si-PPARδ detected by Western blot (n = 3). **C** PPARγ, COL I, COL III, and α-SMA protein expression within HSFs after being treated with si-YBX1#1 or/and si- PPARγ detected by Western blot (n = 3). Sample size is indicated as individual plots in column graphs. Results are indicated by mean ± SD. $^{*}P < 0.05$, $^{**}P < 0.01$.

Supplementary Methods

Chromatin immunoprecipitation (ChIP) assay

Lysates obtained from HSFs were utilized in ChIP assay using SimpleChIP® Enzymatic Chromatin IP Kit SimpleChIP Plus Sonication Chromatin IP Kit (#56383, CST, Danvers, MA, USA) according to specific protocol. After collection of cell samples, RNase A (ST577, Beyotime, China) was applied to exclude RNA interference. Briefly, primary antibody and isotype control IgG antibody (Antibodies utilized in ChIP were provided in Antibodies and Reagents section) were added for immunoprecipitation. Through phenol–chloroform extraction and ethanol precipitation, we purified immunoprecipitated DNA and starting DNA extracted from cell lysate aliquots after RNase A and proteinase K treatment. qRT-PCR was carried out to examine the purified genomic DNA. We determined % of Input through $100\% \times 2(^{-\triangle CT})$, and $^{\triangle}CT$ = Ct (Ip) – Ct (Input). Used primers were as follows: Human PPARα: Forward 5’- GTTTTCTCTCCCTAAAACCTTGGG-3’ and Reverse 5’-ACCTCCGGGCTCAAAGACA-3’; Human PPARδ: Forward 5’-GTGAGGAGCCTCTCCGCCCGG-3’ and Reverse 5’-TGGCCCGTTCTCAATGAGCTGTT-3’; Human PPARγ: Forward 5’-GCTGAGATTACAGGCACGTG-3’ and Reverse 5’-GTATGGCAGCTCACGCCTGTAATC-3’.
