# Supplementary material for: CILP1 interacting with YBX1 promotes hypertrophic scar formation by suppressing PPARs transcription
Source: Cell Death Dis. 2025 May 9;16(1):371. doi: 10.1038/s41419-025-07554-8 (PMC12064789; doi:10.1038/s41419-025-07554-8)

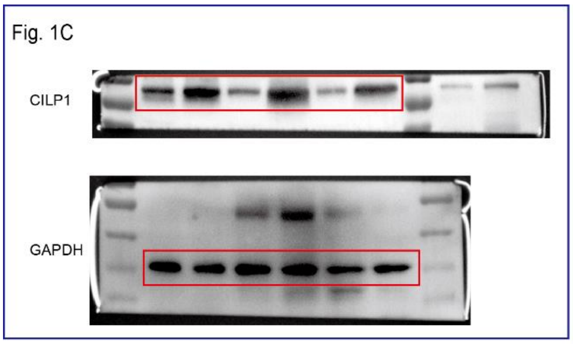
Fig. 1C
CILP1
GAPDH

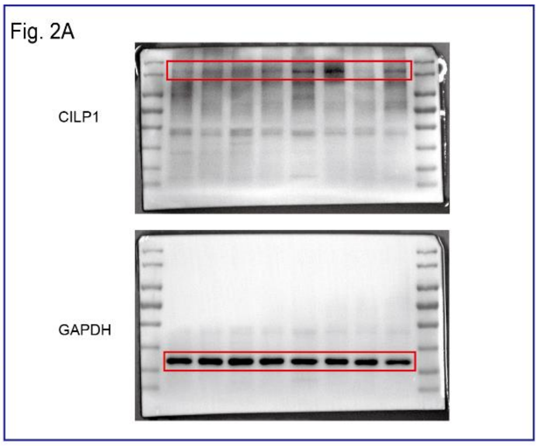
Fig. 2A
CILP1
GAPDH

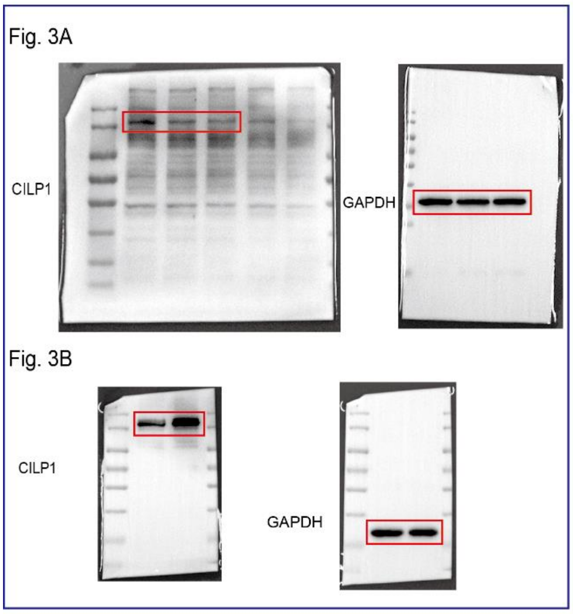
Fig. 3A
CILP1
GAPDH
Fig. 3B
CILP1
GAPDH

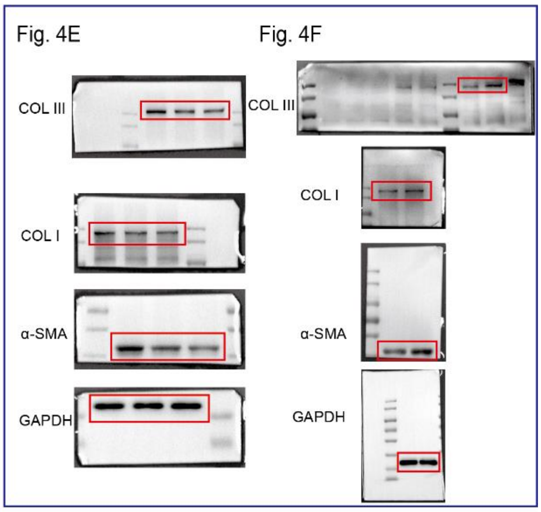
Fig. 4E
COL III
COL I
α-SMA
GAPDH
Fig. 4F
COL III
COL I
α-SMA
GAPDH

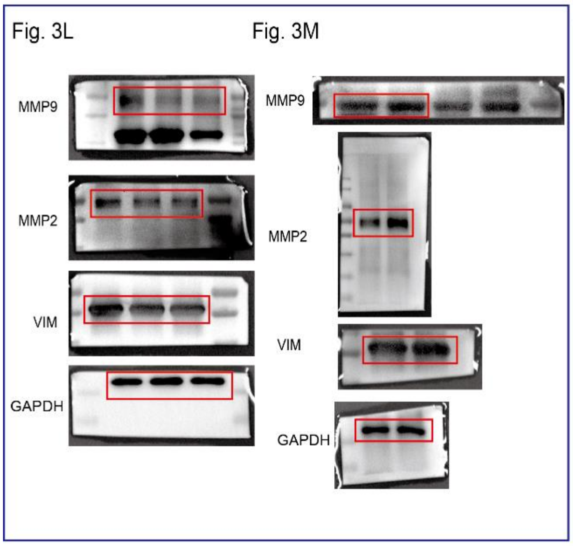
Fig. 3L
MMP9
MMP2
VIM
GAPDH
Fig. 3M
MMP9
MMP2
VIM
GAPDH

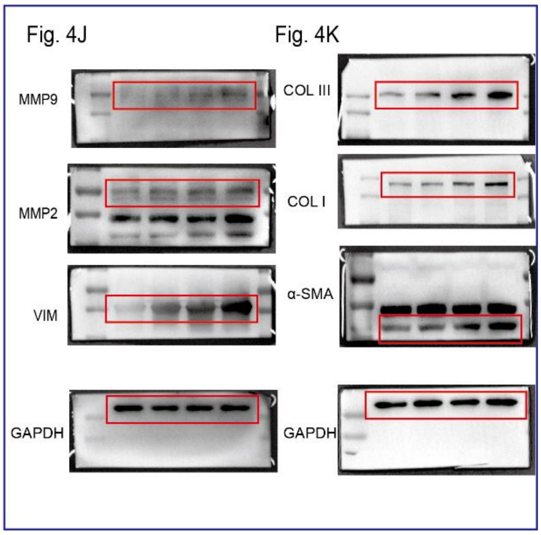
Fig. 4J
MMP9
MMP2
VIM
GAPDH
Fig. 4K
COL III
COL I
α-SMA
GAPDH

Fig. 5A

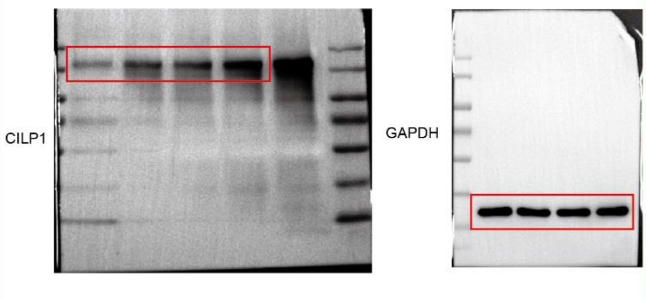

Fig. 5E

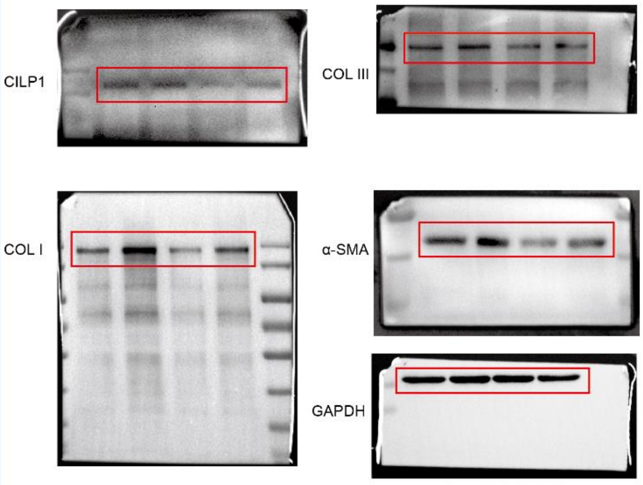

Fig. 5F

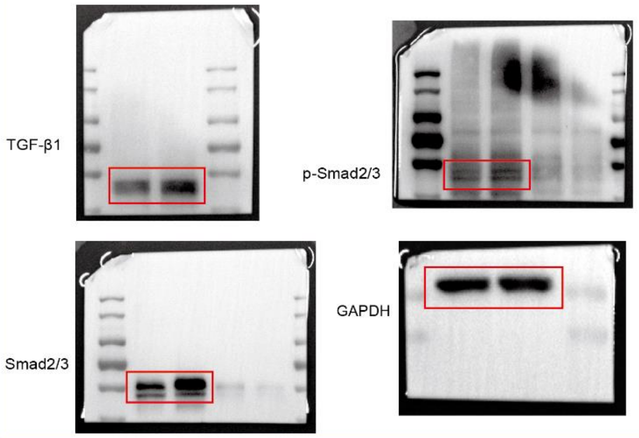

Fig. 5G

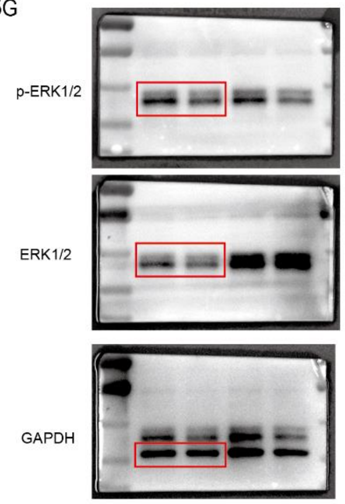

Fig. 5J

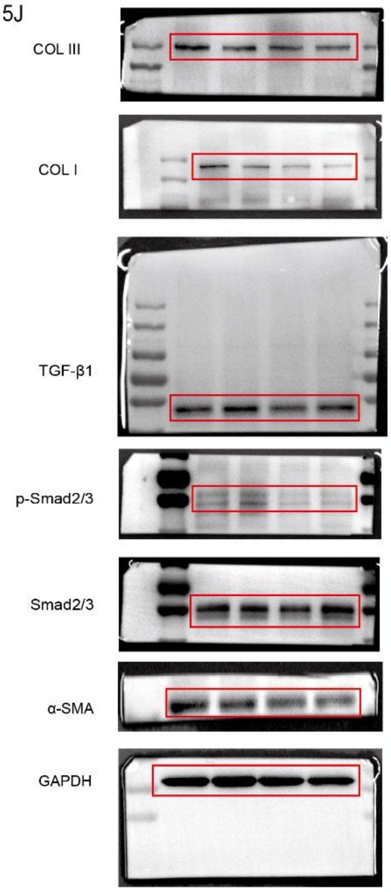

Fig. 6D

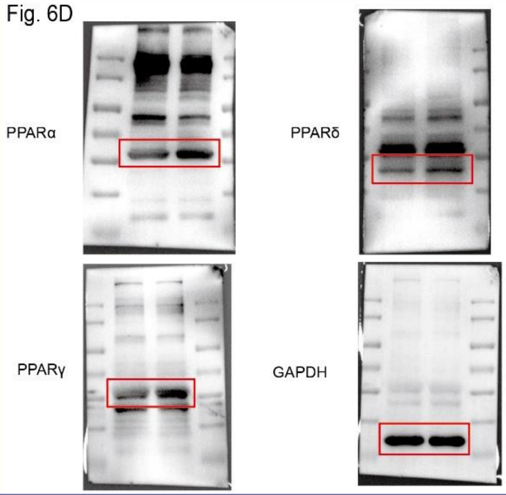

Fig. 7D

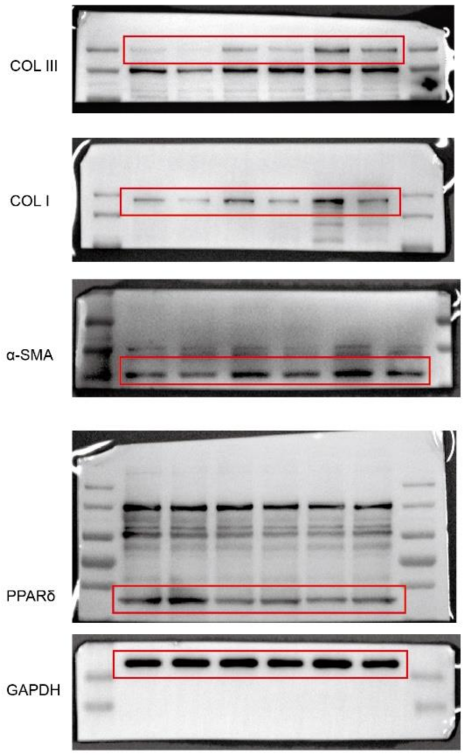

Fig. 7E

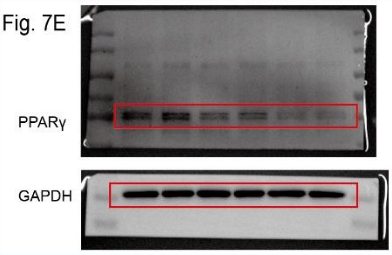

Fig. 7C

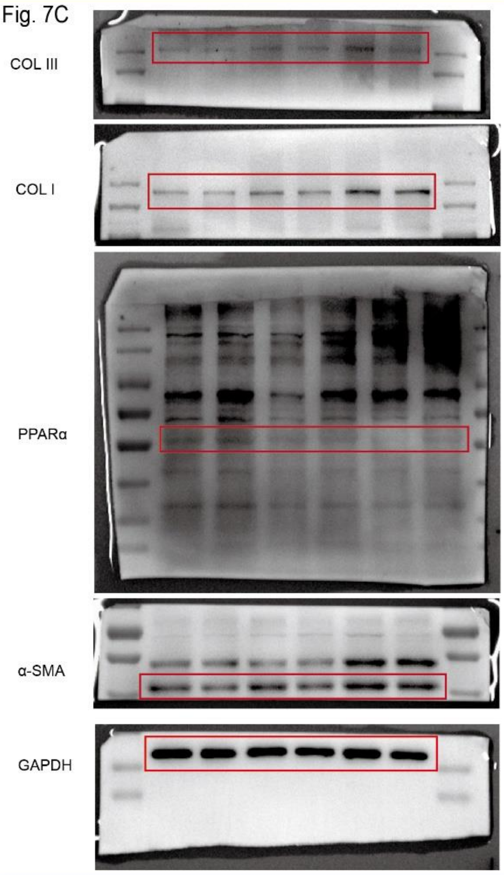

Fig. 7E

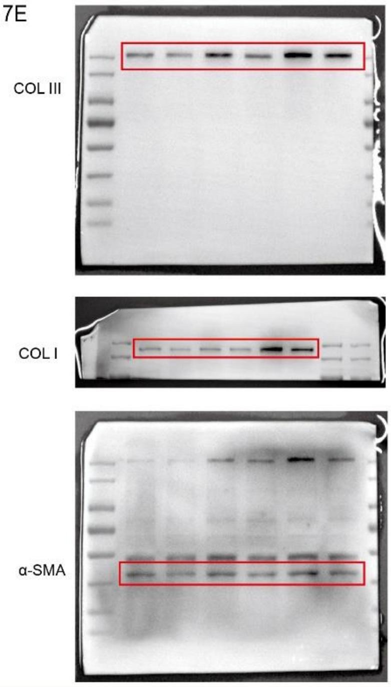

Fig. 7D

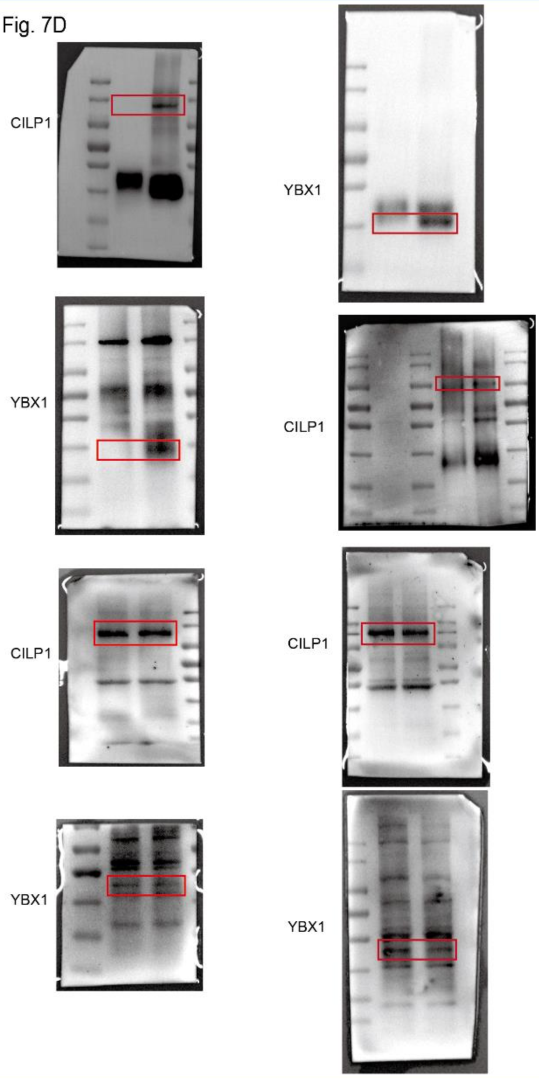

Fig. 7E

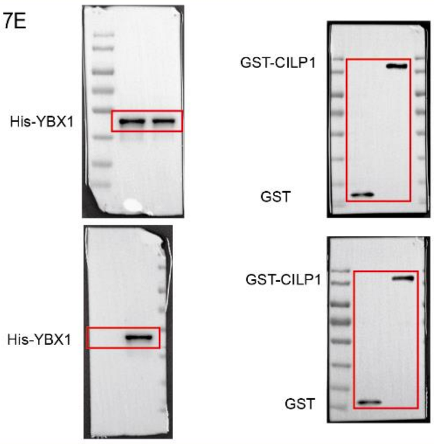

Fig. 7F

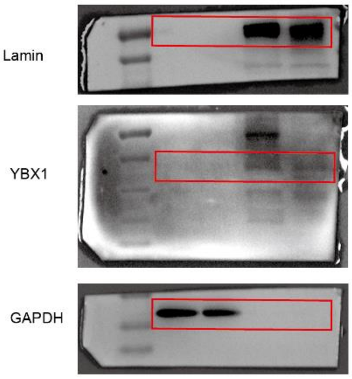

Fig. 7H

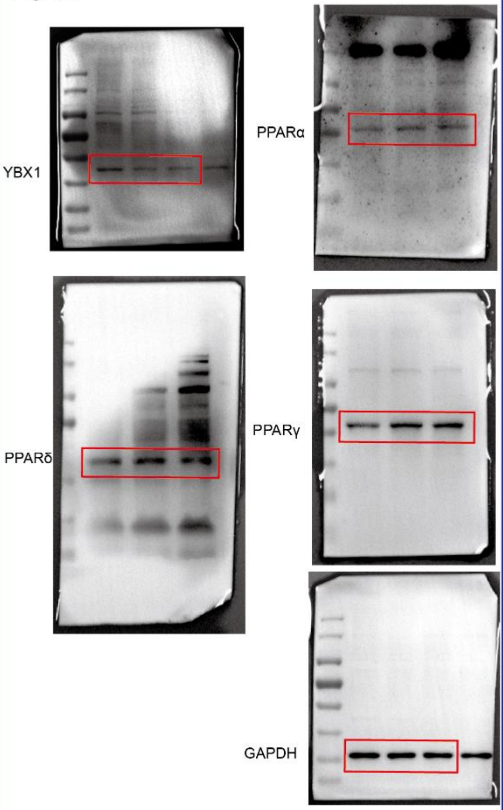

Fig. 9A

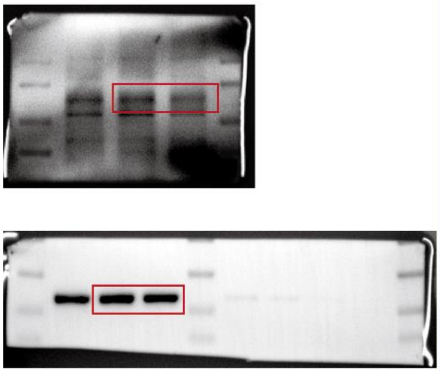

Fig. 9J

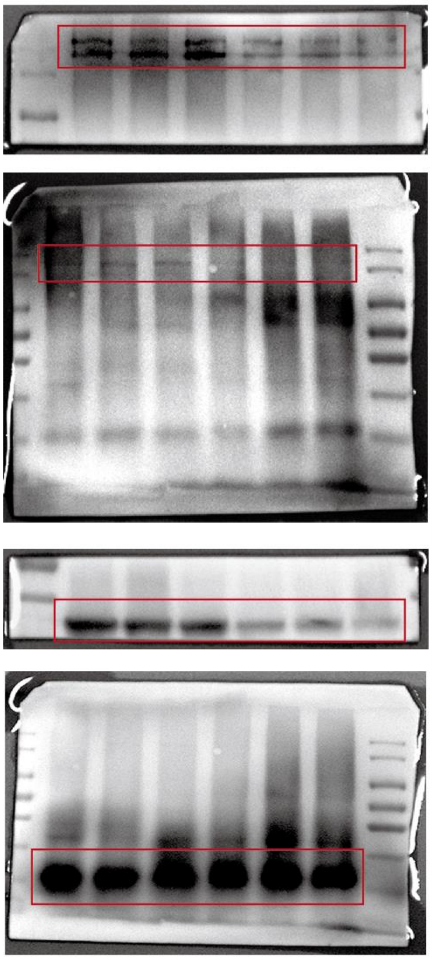

Fig. 10I

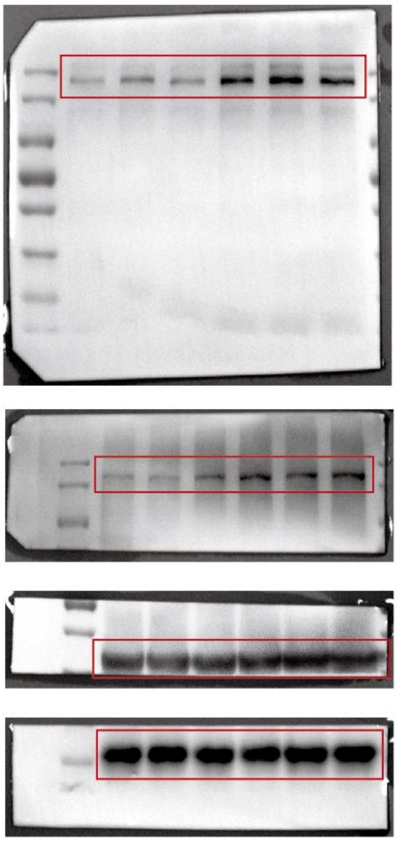

Supplementary Fig. 1A

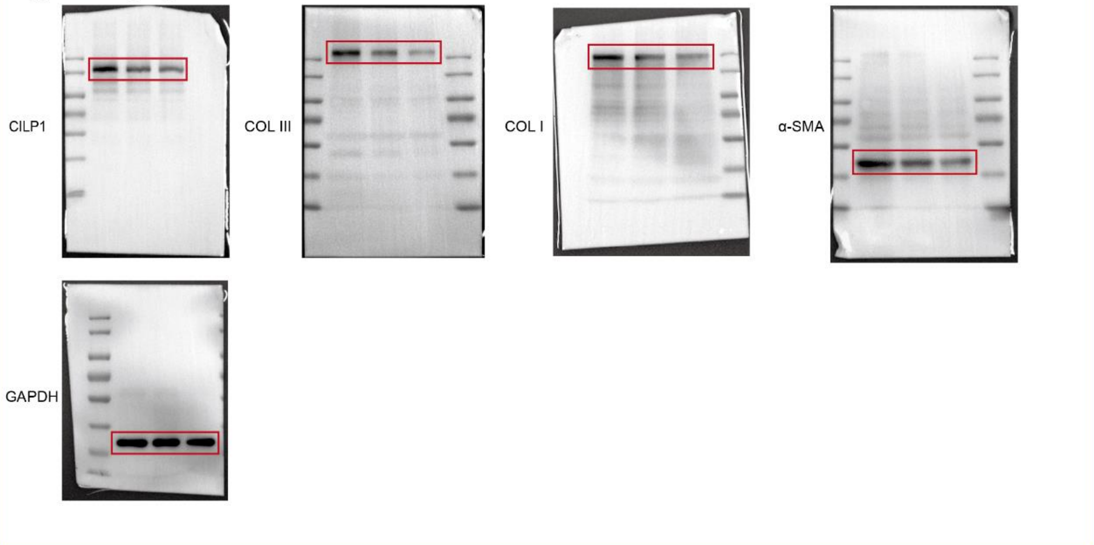

Supplementary Fig. 1B

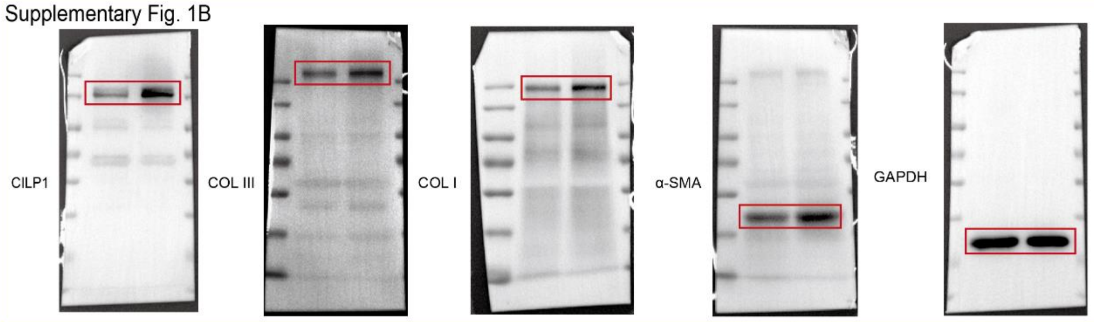

Supplementary Fig. 1C

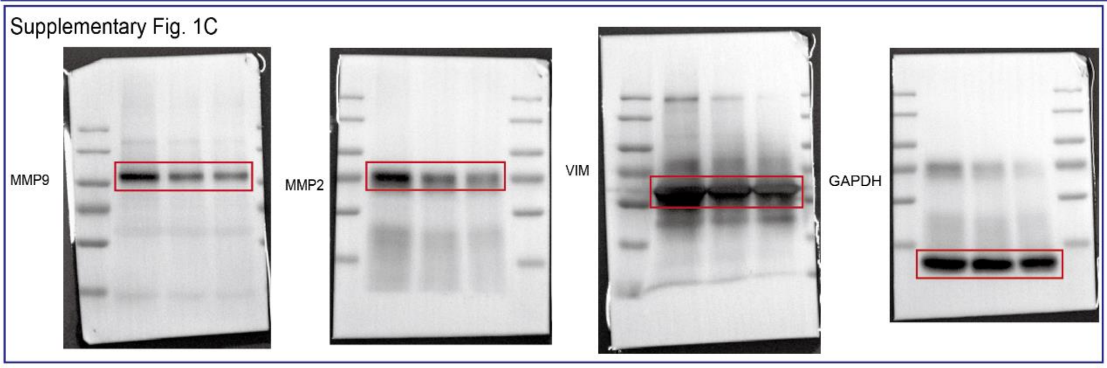

Supplementary Fig. 1D

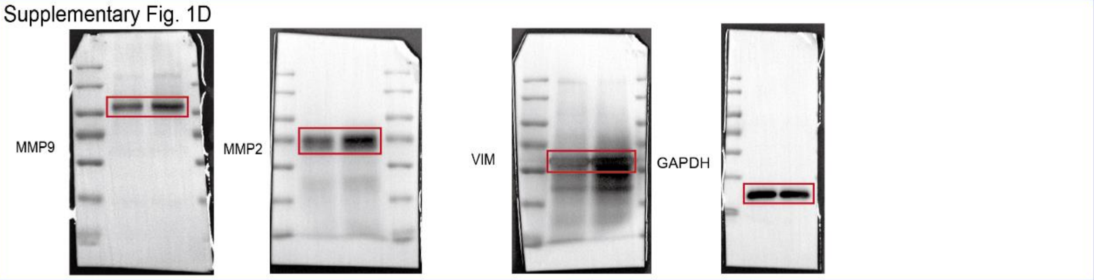

Supplementary Fig. 2B

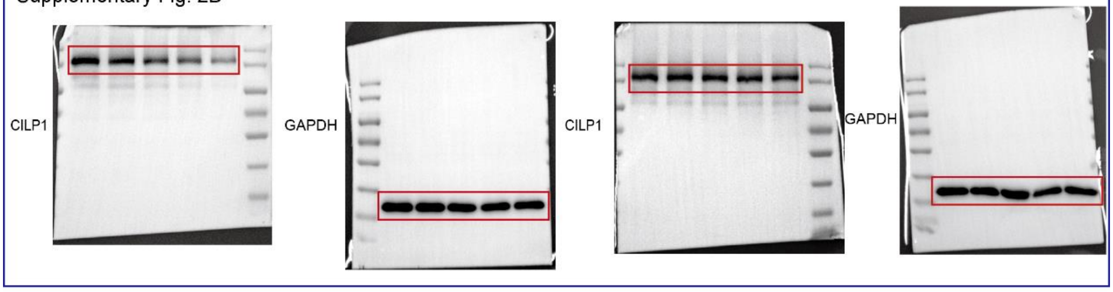

Supplementary Fig. 3A

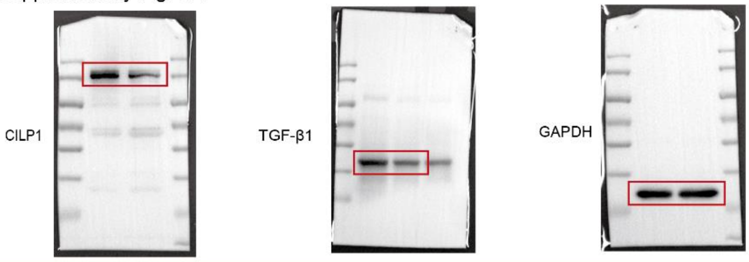

Supplementary Fig. 3C

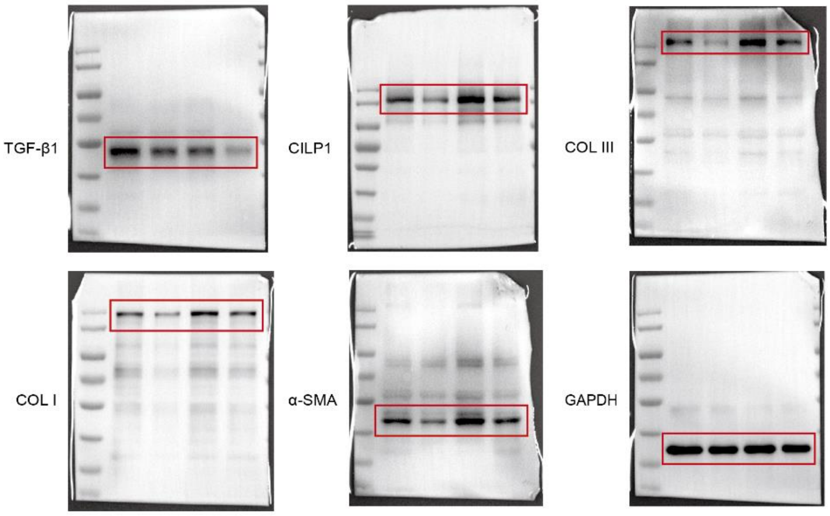

Supplementary Fig. 4

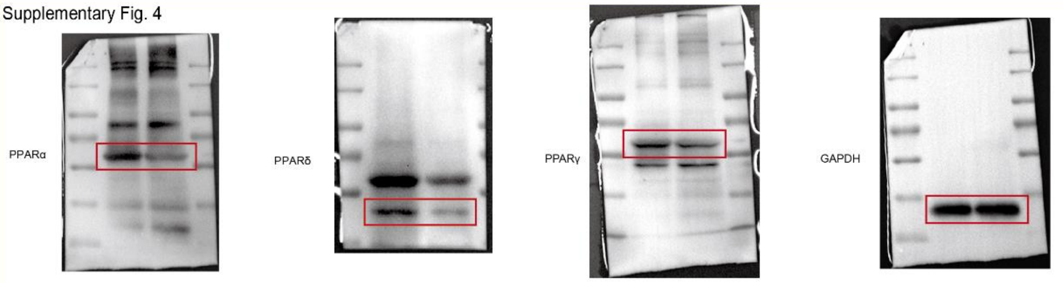

Supplementary Fig. 5

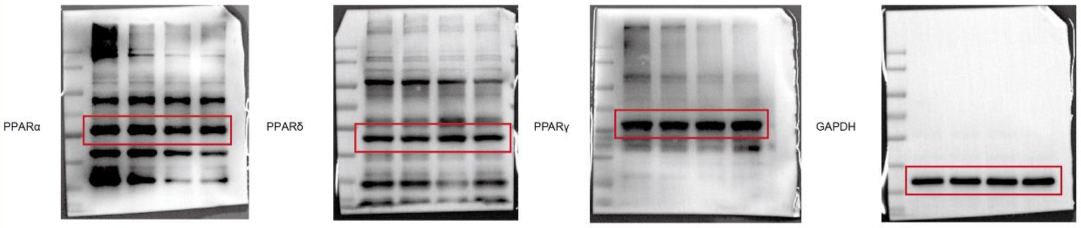

Supplementary Fig. 6A

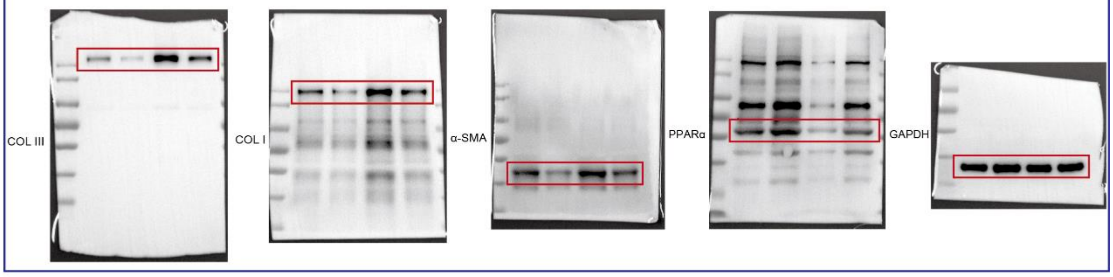

Supplementary Fig. 6B

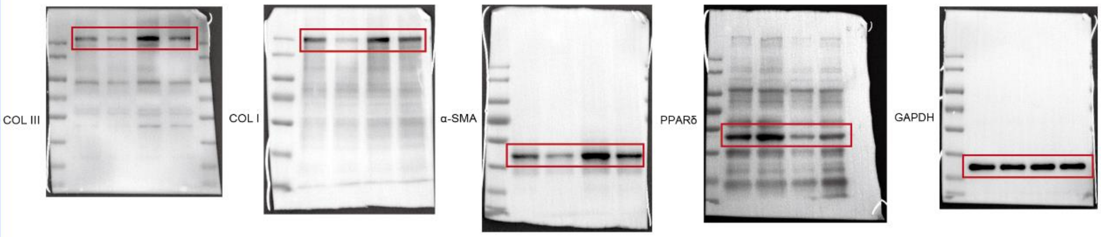

Supplementary Fig. 6C

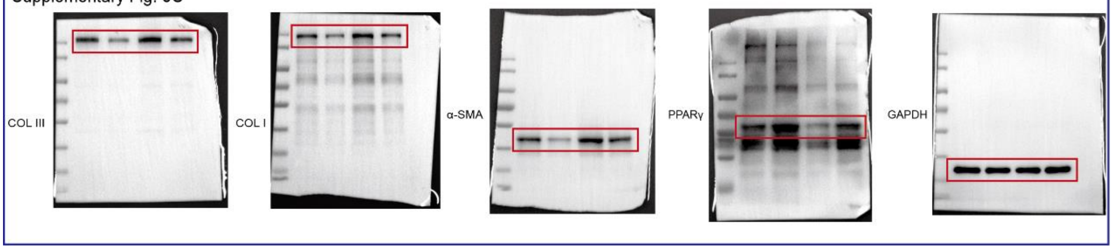

Supplementary Fig. 7C

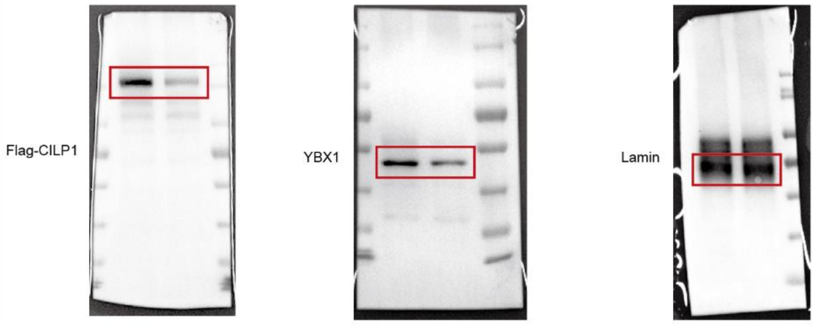

Supplementary Fig. 7C

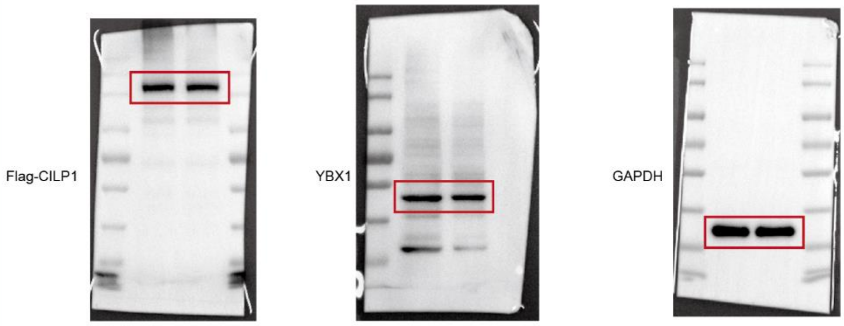

Supplementary Fig. 7D

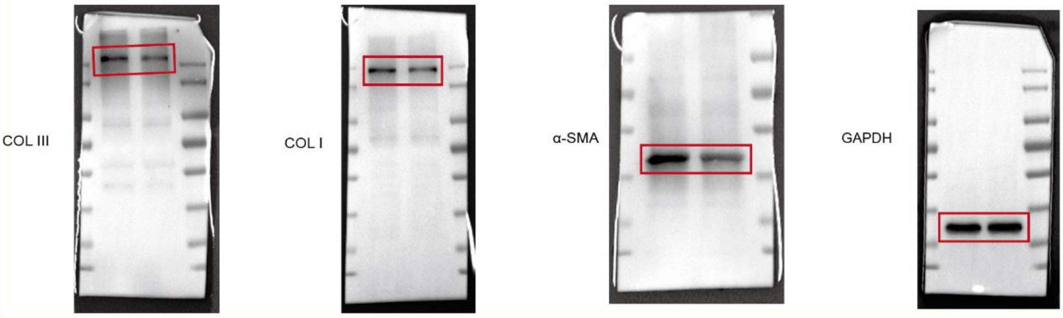

Supplementary Fig. 9A

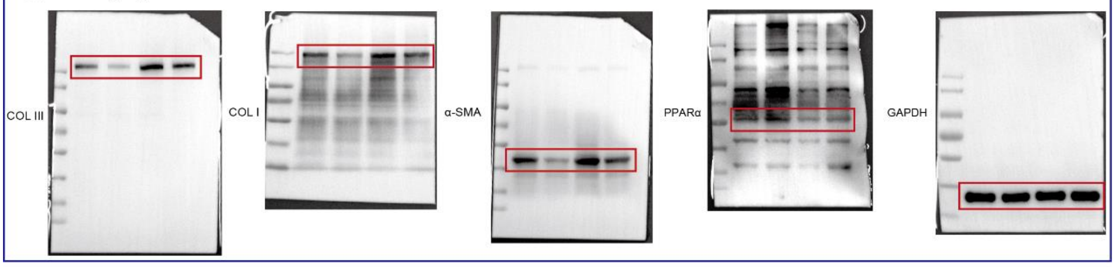

Supplementary Fig. 9B

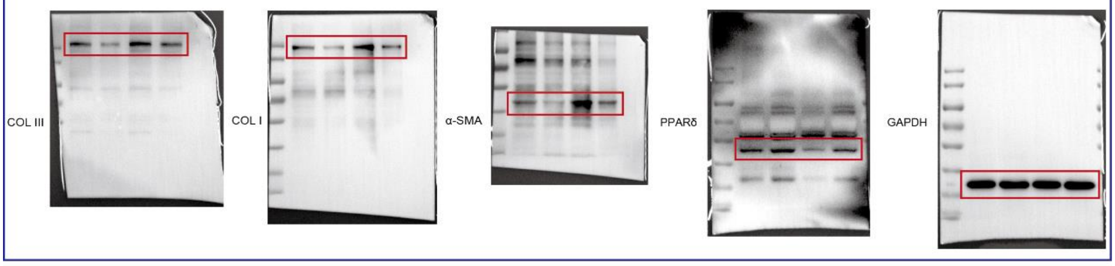

Supplementary Fig. 9C

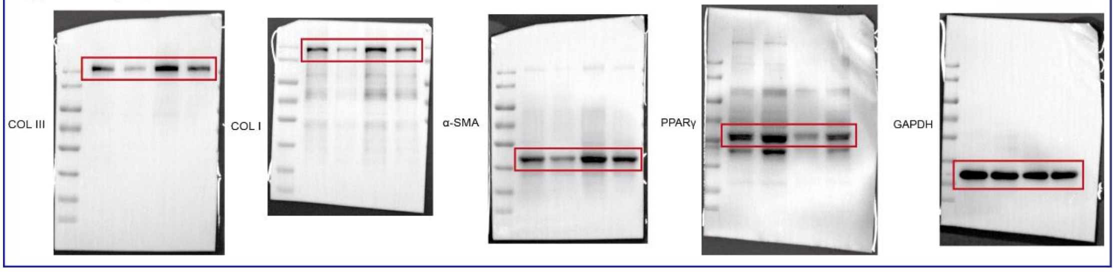

Supplement: Supplementary file 2 — Full and uncropped western blots [file 41419_2025_7554_MOESM2_ESM.pdf]
